# Demographic history and adaptive synonymous and nonsynonymous variants of nuclear genes in *Rhododendron oldhamii* (Ericaceae)

**DOI:** 10.1038/s41598-020-73748-z

**Published:** 2020-10-07

**Authors:** Yi-Chiang Hsieh, Chung-Te Chang, Jeng-Der Chung, Shih-Ying Hwang

**Affiliations:** 1 School of Life Science, National Taiwan Normal University, 88 Tingchow Road, Section 4, Taipei, 11677 Taiwan; 2 Department of Life Science, Tunghai University, 1727 Taiwan Boulevard, Section 4, Taichung, 40704 Taiwan; 3 Division of Silviculture, Taiwan Forestry Research Institute, 53 Nanhai Road, Taipei, 10066 Taiwan

**Keywords:** Evolution, Genetics

## Abstract

Demographic events are important in shaping the population genetic structure and exon variation can play roles in adaptive divergence. Twelve nuclear genes were used to investigate the species-level phylogeography of *Rhododendron oldhamii,* test the difference in the average GC content of coding sites and of third codon positions with that of surrounding non-coding regions, and test exon variants associated with environmental variables. Spatial expansion was suggested by *R*_2_ index of the aligned intron sequences of all genes of the regional samples and sum of squared deviations statistic of the aligned intron sequences of all genes individually and of all genes of the regional and pooled samples. The level of genetic differentiation was significantly different between regional samples. Significantly lower and higher average GC contents across 94 sequences of the 12 genes at third codon positions of coding sequences than that of surrounding non-coding regions were found. We found seven exon variants associated strongly with environmental variables. Our results demonstrated spatial expansion of *R. oldhamii* in the late Pleistocene and the optimal third codon position could end in A or T rather than G or C as frequent alleles and could have been important for adaptive divergence in *R. oldhamii*.

Spatial and temporal patterns underlie population demographic processes of plant species and the phylogenetic relationship between and within species can be revealed by using chloroplast and nuclear DNA sequence data^1–3^. Molecular techniques, such as amplified fragment length polymorphisms (AFLPs), expressed sequence tag simple sequence repeats (EST-SSRs), and methylation-sensitive amplification polymorphisms (MSAPs) have been commonly employed in investigation testing for environmentally dependent local adaptation^4–6^. Nonetheless, nuclear gene sequences can be amplified and sequenced spanning coding and non-coding regions. The variation in non-coding and coding sequences in nuclear genes can be used, respectively, in investigation of phylogeny and phylogeography and in testing for correlation with environmental variables contributing to adaptive evolution^4,7,8^. Within the coding region of a gene, the ratio of the number of nonsynonymous substitutions per nonsynonymous site to the number of synonymous substitutions per synonymous site is commonly used for the inference of adaptive evolution of genes driven by natural selection^9–11^, and synonymous substitutions are thought to be inconsequential because of the conservative nature of amino acids. However, synonymous substitutions can have significant effects on gene expression, protein folding, and protein cellular function^12,13^, and hence synonymous substitutions may not be "silent"^14^.

The difference in the relative frequency of synonymous codons for individual amino acids in protein coding sequences is coined as codon usage bias. Codon bias can vary among species and/or among genes of a genome and may be derived via mutational bias processes, GC biased conversion, or driven by selection co-adapting with tRNAs in optimizing the efficiency and accuracy of translation^15–17^. Theory suggests that the strength of natural selection on synonymous sites may be weak and effective population size is thought to be the determining factor for natural selection to be effective on codon usage pattern^18,19^, and synonymous substitution is subjected to strong purifying selection^20,21^. Nonetheless, selection acting on codon bias has been found in prokaryotes with large effective population sizes^22,23^ and in eukaryotic species with low effective population sizes^24–26^.

In genes with high codon bias, the "preferred codons" often end in either C or G according to major codon preference model^27,28^. The level of GC content at third codon positions is considered as an indicator reflecting codon usage pattern, and the level of gene expression has been found to be positively correlated with the level of GC content at third codon positions^29,30^. Studies have shown that overall codon bias is more phenomenal in monocots than in dicots and, respectively, tended to use C/G and A/T at third codon positions^31,32^. However, optimal codons tend to end in G or C in dicots, resulting in higher average GC content at coding sites and at third codon positions compared with surrounding non-coding regions^32,33^.

*Rhododendron oldhamii* Maximowicz belongs to the subgenus *Tsutsusi* of Ericaceae is an endemic dicot species widely, but fragmented distributed in the lowlands and up to 2,500 m in the humid understory of broadleaf forests in Taiwan. Heterogeneity in flowering times of *R. oldhamii* populations located in different geographic areas, across the species’ distribution range, have been shown either via examination of herbarium specimen records^34^ or by field studies^35^. Differential flowering times of species distributed in different geographic areas can result in reproductive isolation or reproductive incompatibility^36^, and in consequence a limited gene flow between populations^36,37^. Hsieh et al.^4^ showed that gene dispersal was limited within geographic regions of *R. oldhamii* and inferred that the discontinuities of population distribution resulted from recent population bottlenecks during the Holocene based on EST-SSR data. Although organisms in isolated, small populations may be restricted in developing local adaptation in response to changing environments^38^, study based on EST-SSR found population divergence at regional level in association with environmental variables underlying local adaptation in *R. oldhamii*^4^. Moreover, genetic and epigenetic variations based on AFLP and MSAP also found environmentally dependent adaptive divergence in populations of *R. oldhamii*^5^. *R. oldhamii* discontinuously distributed in a wide geographic range makes it an excellent exemplar endemic subtropical forest tree species occurring in Taiwan for investigating not only the population divergence associated with environmental differences, but also the phylogeographic history related to the current genetic structure of this species.

Since it has been shown that adaptive divergence driven by natural selection at local scales in *R. oldhamii* based on EST-SSRs^4^, it is worthwhile to study further from the evolutionary perspective of natural selection on codon usage bias using DNA coding sequences. Additionally, the far-distant past demographic history shaping the genetic structure and distribution of *R. oldhamii* can be inferred using nuclear non-coding sequences in contrast to using frequency data of EST-SSRs reflecting more recent demography in the previous study^4^. Strong correlations of synonymous and nonsynonymous substitutions with environmental variables using regression approaches^7,8,39,40^ may provide as evidence suggesting that they would have experienced the effects of selection. We hypothesized that both synonymous and nonsynonymous substitutions of nuclear coding sequences, particularly synonymous substitutions at third codon positions, in natural populations of *R. oldhamii* (Fig. 1) might have driven by natural selective forces in association with environmental heterogeneity. Using partial genomic DNA sequences of 12 haphazardly selected nuclear genes (Supplementary Table S1), we aimed to (1) investigate the species-level phylogeographic history of *R. oldhamii* based on intron sequences of multiple nuclear gene loci, (2) test the significant differences in the average GC content of coding sites and of third codon positions of nuclear coding sequences with that of surrounding non-coding regions, and (3) test the associations of synonymous and nonsynonymous variants in coding sequences with environmental variables.

**Figure 1:**
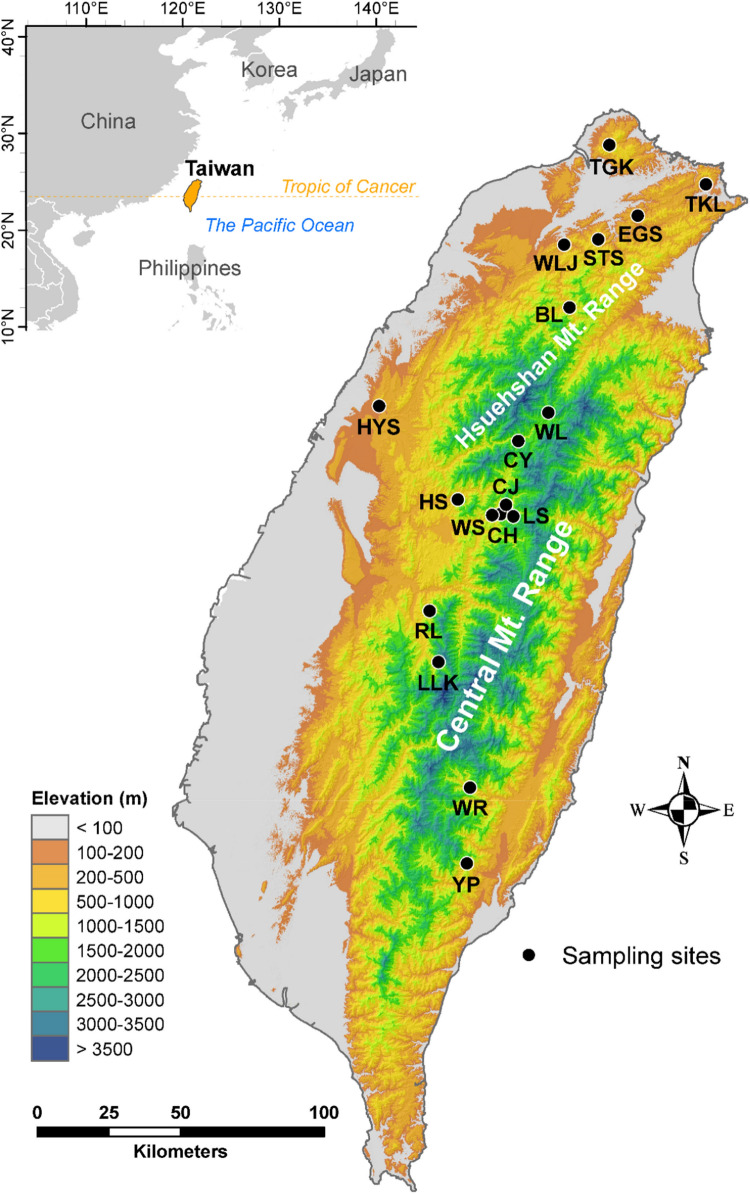
Sample locations of the 18 populations of *Rhododendron oldhamii* distributed in Taiwan. The 18 populations were assigned to four geographic regions according to EST-SSR clustering (Table 1)^4^. The four geographic groups were north group (populations BL, EGS, HYS, STS, TGK, TKL, and WLJ), central group (population WL), south group (populations CH, CJ, CY, HS, LLK, LS, RL, and WS), and southeast group (populations WR and YP). See Table 1 for population code abbreviations. We generated the map using ArcGIS v.10.6. The ASTER GDEM (Global Digital Elevation Model; https://asterweb.jpl.nasa.gov/gdem.asp) is used for elevational background.

## Results

### Genetic diversity

The sequences of 12 nuclear DNA loci (Supplementary Table S1) were obtained from 47 individuals across 18 populations (four geographic regions^4^) (Table 1). The length of aligned sequences of the pooled samples, including exon and intron sequences, for each locus ranged from 509 bp (*GAPC1*) to 908 bp (*PCFS4*) (Supplementary Table S2), and the total aligned length was 8871 bp. The total aligned length for exon and intron, respectively, was 2082 bp and 6798 bp. Lengths of exons and introns of the 12 genes, respectively, ranged from 36 bp (*GRP7*) to 372 bp (*LACS8*) and from 280 bp (*LHCA1*) to 859 bp (*GRP7*). The minimum number of recombination events (*R*_m_) within gene ranged from 0 (*CPD*) to 10 (*GAPC1*) (Table 2). The number of haplotypes ranged from 2 to 16 and from 16 to 30, respectively, for the 18 populations and the four geographic regions. The number of haplotypes for the total aligned sequences was 94 (Table 1). Nucleotide diversity *π* ranged from 0.00163 (population CJ) to 0.00785 (population EGS) and from 0.00586 (southeast) to 0.00659 (central), respectively, for the 18 populations and the four geographic regions. Waterson’s nucleotide diversity measure, *θ*_*w*_, based on segregating sites ranged from 0.00163 (population CJ) to 0.00785 (population EGS) and from 0.00552 (south) to 0.00695 (central) for the 18 populations and the four geographic regions, respectively. The nucleotide polymorphisms (*π* and *θ*_w_) for individual gene in each population were reported in Supplementary Tables S3 and S4. Friedman test revealed no significant difference in regional comparisons neither in *π* nor in *θ*_*w*_ (*χ*^2^ = 0.7, *P* = 0.873 in both cases). However, significant differences of *π* and *θ*_*w*_ among populations were found (*χ*^2^ = 35.10, *P* = 0.006; *χ*^2^ = 35.33, *P* = 0.0056, respectively). Moreover, significant differences of *π* and *θ*_*w*_ were found in pairwise population comparisons after correction for multiple comparisons at false discovery rate (FDR) of 5%, but no significant difference was found in between region comparisons (Supplementary Table S5). Significant positive inbreeding coefficient (*F*_IS_), indicative of departure from Hardy–Weinberg equilibrium, representing homozygote excess, was found for all populations that were estimable (sample size > 1) except the population LLK (Table 1).

**Table 1:** Population information and number of haplotypes, nucleotide diversity, and inbreeding coefficients (*F*_IS_) based on the total aligned intron sequences of 12 nuclear genes for the 18 *Rhododendron oldhamii* populations.

Population (code)	Region	*N*	Locality (°E/°N)	*N*_*h*_	*π* (SD)	*θ*_*w*_ (SD)	*F*_IS_ (95% CI)
**Population**							
Baling (BL)	North	4	121.38/24.68	8	0.00596 (0.00067)	0.00554 (0.00243)	**0.097** (0.025, 0.169)
Ergirshan (EGS)	North	1	121.62/24.97	2	0.00785 (0.00392)	0.00785 (0.0056)	–
Huoyanshan (HYS)	North	5	120.73/24.37	10	0.00549 (0.00056)	0.00530 (0.00218)	**0.423** (0.339, 0.508)
Shihtoushan (STS)	North	1	121.48/24.89	2	0.00563 (0.00281)	0.00563 (0.00403)	–
Tsaigongkeng (TGK)	North	1	121.52/25.19	2	0.00370 (0.00185)	0.00370 (0.00267)	–
Tsanguanliao (TKL)	North	1	121.86/25.06	2	0.00399 (0.002)	0.00399 (0.00288)	–
Wuliaojian (WLJ)	North	1	121.37/24.88	2	0.00429 (0.00215)	0.00429 (0.00309)	–
Wuling (WL)	Central	8	121.31/24.35	16	0.00668 (0.001)	0.00699 (0.00253)	**0.670** (0.619, 0.721)
Chungheng (CH)	South	1	121.15/24.03	2	0.00341 (0.00171)	0.00341 (0.00246)	–
Chingjing (CJ)	South	1	121.16/24.06	2	0.00163 (0.00081)	0.00163 (0.0012)	–
Chiayang (CY)	South	3	121.21/24.26	6	0.00681 (0.00096)	0.00650 (0.0031)	**0.340** (0.267, 0.414)
Hueisun (HS)	South	2	121.00/24.08	4	0.00655 (0.00174)	0.00671 (0.00366)	**0.133** (0.033, 0.234)
Leleku (LLK)	South	4	120.93/23.56	8	0.00613 (0.00069)	0.00555 (0.00243)	− 0.026 (− 0.095, 0.044)
Lushan (LS)	South	2	121.19/24.02	4	0.00733 (0.00185)	0.00729 (0.00397)	**0.116** (0.012, 0.220)
Renluen (RL)	South	1	120.90/23.73	2	0.00237 (0.00119)	0.00237 (0.00173)	–
Wushe (WS)	South	1	121.12/24.03	2	0.00756 (0.00378)	0.00756 (0.0054)	–
Wuru (WR)	Southeast	5	121.04/23.17	10	0.00506 (0.00071)	0.00507 (0.00209)	**0.191** (0.115, 0.266)
Yeinping (YP)	Southeast	5	121.03/22.93	10	0.00578 (0.00074)	0.00561 (0.00231)	**0.265** (0.176, 0.354)
**Region**							
North		14		28	0.00594 (0.00032)	0.00626 (0.002)	
Central		8		16	0.00659 (0.001)	0.00695 (0.00252)	
South		15		30	0.00605 (0.00034)	0.00552 (0.00174)	
Southeast		10		20	0.00586 (0.00043)	0.00606 (0.00209)	
Total		47		94	0.00659 (0.00027)	0.00945 (0.00235)	

*N*, sample size; *N*_h_, number of haplotypes; *π*, the average number of pairwise nucleotide differences per site; *θ*_*w*_, the average nucleotide diversity of segregating site; *F*_IS_, inbreeding coefficients.

*F*_IS_ values do not bracket zero are in bold.

Classification of populations into different geographic regions was based on the results of a previous study^6^.

**Table 2:** Summary of nucleotide polymorphism and neutrality tests based on the aligned intron sequences of individual genes and the total aligned intron sequences for *Rhododendron oldhamii*.

Locus	Intron aligned length (bp)	*Rm*	*S*	*π*	*θ*_w_	*Hd*	*Nh*	Neutrality test				
								*D*	D*	F*	*R*_*2*_	SSD
*AMP1*	486	1	51	0.01059	0.03925	0.669	14	− **2.377**	1.348	− 0.191	**0.026**	0.00209
*ATMYB33*	695	2	24	0.00137	0.00689	0.427	16	− **2.394**	− **3.566**	− **3.732**	**0.023**	0.00143
*CPD*	727	0	13	0.00117	0.00351	0.641	14	− **1.810**	− 1.430	− 1.863	**0.033**	0.00207
*GAPC1*	356	10	26	0.01656	0.01456	0.732	22	0.413	0.777	0.763	0.110	0.01913
*GRP7*	859	1	26	0.00123	0.00593	0.578	21	− **2.427**	− 1.648	− **2.329**	**0.023**	0.00141
*HEME2*	633	4	41	0.01673	0.01270	0.830	28	0.999	− 1.256	− 0.432	0.126	0.02930
*LACS8*	332	1	11	0.00507	0.00672	0.713	14	− 0.645	− 1.197	− 1.192	0.072	0.02205
*LHCA1*	280	2	12	0.00372	0.00850	0.452	10	− 1.506	0.230	− 0.457	0.043	0.00329
*PCFS4*	830	5	32	0.00378	0.00763	0.875	27	− 1.558	− 1.454	− 1.790	**0.047**	0.00111
*PMDH2*	538	8	27	0.01046	0.01021	0.905	29	− 0.140	− 0.090	− 0.131	0.098	0.00823
*SPA1*	503	1	20	0.00527	0.00779	0.782	15	− 0.943	0.418	− 0.107	0.065	0.01036
*SUI1*	550	7	30	0.01287	0.01076	0.712	26	0.172	− 0.084	0.020	0.115	0.01506
**Region**												
North		24	164			1.000	28	− 0.265	− 0.280	− 0.325	0.112	0.00330
Central		11	150			1.000	16	− 0.359	0.203	0.049	0.127	0.01023
South		34	147			1.000	30	0.343	0.183	0.280	0.131	0.00381
Southeast		14	145			1.000	20	− 0.134	− 0.041	− 0.081	0.122	0.00616
Total	6798	51	313			1.000	94	− 1.094	− 0.750	− 1.079	**0.066**	0.00123

*Rm*, minimum number of recombination events; *S*, number of segregating sites; *π*, the average number of pairwise nucleotide diversity per site; *θ*_w_, the average nucleotide diversity of segregating site; *Hd*, haplotype diversity; *Nh*, number of haplotypes. *D*, Tajima's *D*; D*, Fu & Li's D*; F*, Fu & Li's F*; SSD, sum of square deviations.

P values of neutrality tests < 0.05 are in bold.

### Demography and genetic structure

Neutrality test statistics including Tajima’s *D* and Fu and Li’s D* and F* were mostly negative, albeit not significant, based on the aligned intron sequences of the 12 loci individually and the total aligned intron sequences of the pooled samples (Table 2). Consistent significant negative values of these statistics were only found for *ATMYB33*. Significant small *R*_2_ values were found for the aligned intron sequences of the *AMP1*, *ATMYB33*, *CPD*, *GRP7*, and *PCFS4* genes of the pooled samples and for the total aligned intron sequences of the pooled samples. However, spatial expansion model was rejected by neutrality test statistics including *D*, D*, F*, and *R*_2_ based on the total aligned intron sequences of the regional samples. Nonetheless, non-significant sum of square deviations (SSD) estimation revealed that the spatial expansion model could not be rejected based on the aligned intron sequences of the pooled samples of the 12 loci individually. Spatial expansion was also suggested by SSD when analyzed using the total aligned intron sequences of the regional and the pooled samples. Comparable sample sizes among regions and approximately equal migration rates among populations within regions were found based on the goodness-of-fit test for mismatch distribution under spatial expansion model (Supplementary Table S6).

The level of genetic differentiation was found to be significant when compared among regions (*F*_ST_ = 0.074, *P* = 0.001), but not significant when compared among populations (*F*_ST_ = -0.0078, *P* = 0.331). Significant pairwise *F*_ST_ was also found for between region comparisons (Supplementary Table S7). Additionally, genetic clustering using discriminant analysis of principal components (DAPC) showed no clear population or regional distinction (Supplementary Fig. S1).

### The average GC content

The average GC content at coding sites, at third codon positions, and in surrounding non-coding regions across 94 sequences of the 12 genes were 0.453, 0.419, and 0.356, respectively (Table 3). Paired Wilcoxon tests found that most of the average GC content at coding sites across 94 sequences of the 12 genes, except *GRP7*, was significantly (*P* < 0001) higher than the average GC content of surrounding non-coding regions (Table 3). Moreover, five (*AMP1, GRP7, LACS8, PCFS4*, and *SPA1*) and seven (*ATMYB33, CPD, GAPC1, HEME2, LHCA1, PMDH2*, and *SUI1*) of the 12 genes, respectively, showed significantly lower and higher average GC content across 94 sequences at third codon positions than that of surrounding non-coding regions.

**Table 3:** Mean GC contents for GC_I_, GC_E_, and GC3_S_ across 94 sequences of the 12 nuclear genes.

Locus	Mean GC content		
	GC_I_	GC_E_	GC3_S_
*AMP1*	0.374	0.434*	0.315^+^
*ATMYB33*	0.308	0.519*	0.385*
*CPD*	0.353	0.429*	0.354*
*GAPC1*	0.351	0.479*	0.632*
*GRP7*	0.384	0.361^+^	0.364^+^
*HEME2*	0.357	0.404*	0.358*
*LACS8*	0.351	0.444*	0.319^+^
*LHCA1*	0.357	0.554*	0.648*
*PCFS4*	0.349	0.500*	0.308^+^
*PMDH2*	0.340	0.457*	0.429*
*SPA1*	0.350	0.396*	0.337^+^
*SUI1*	0.401	0.457*	0.574*
Average	0.356	0.453	0.419

GC_I_, the average GC content of non-coding region.

GC_E_, the average GC content of coding sites.

GC3_S_, the average GC content at third positions of codons.

* and ^+^ represent, respectively, significantly higher and lower GC content based on Wilcoxon paired test (*P* < 0.001). Comparisons between GC_E_ and GC_I_ and between GC3_S_ and GC_I_ were performed.

### Environmental heterogeneity and exon variation explained by environment and geography

We found no environmental heterogeneity among populations based on the eight retained environmental variables using permutational multivariate analysis of variance (PERMANOVA) (*P* = 1). However, significant environmental heterogeneity was found when compared among regions (*F* = 21.52, *R*^2^ = 0.6002, *P* = 0.001). Significant environmental heterogeneity was also found in all pairwise regional comparisons except between the central-south regional group comparison (*P* = 0.334) (Supplementary Table S8).

In the total aligned exon sequences of the pooled samples, 31 exon variable sites were found in nine out of the 12 nuclear genes examined. Using a variation partitioning, the total amount of explainable exon variation (12.13%) was significantly explained by a combinatorial effect of environment and geography (*F* = 1.635, *P* = 0.003), albeit large amount of exon variation was unaccountable (fraction [d]: 87.87%) (Table 4). However, essentially no exon variation was explained purely by geography independent of environment (fraction[c]: adjusted *R*^2^ = − 0.0121, *F* = 0.738, *P* = 0.804). Nevertheless, exon variation was significantly explained by pure environment (fraction[a]: adjusted *R*^2^ = 0.0565, *F* = 1.254, *P* = 0.030), albeit a larger portion of explainable exon variation was attributed to geographically-structured environmental differences (fraction [b]: adjusted *R*^2^ = 0.0769).

**Table 4:** The percentage of exon variation explained by non-geographically-structured environmental variables [a], shared (geographically-structured) environmental variables [b], pure geographic factors [c], and undetermined component [d] analyzed based on the eight retained environmental variables.

	Variation (adjusted * R*^2^)	*F*	*P*
Environment [a]	0.05648	1.3536	0.030
Environment + Geography [b]	0.07694	–	–
Geography [c]	− 0.01211	0.7381	0.804
[a + b + c]	0.12132	1.6351	0.003
Residuals [d]	0.87868	–	–

Proportions of explained variation were obtained from variation partitioning by redundant analysis. *F* and *P* values are specified wherever applicable.

### Associations of environmental variables with exon variant alleles

**Figure 2:**
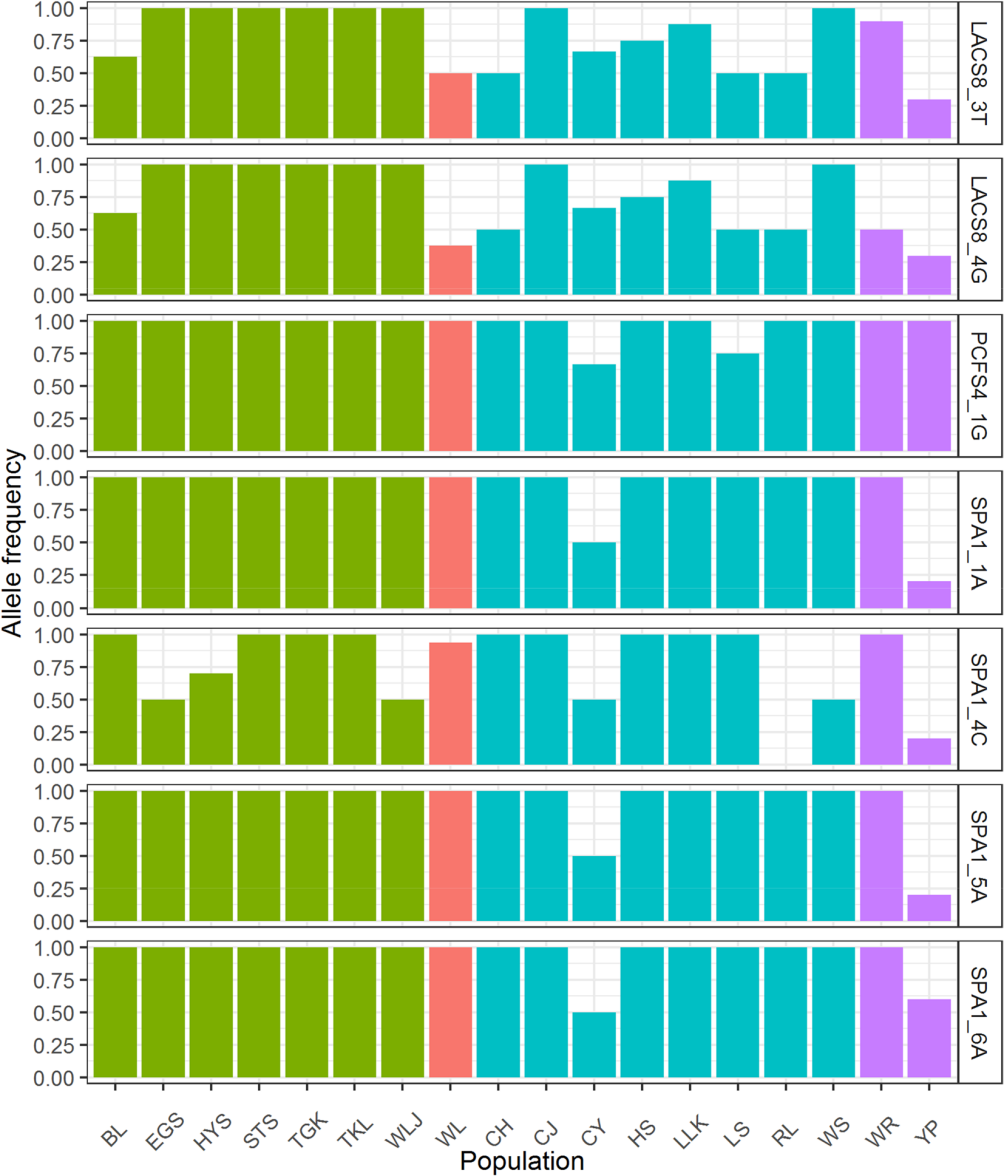
Distributions of frequent allele frequencies of the seven exon variants strongly associated with environmental variables across the 18 *Rhododendron oldhamii* populations.

**Figure 3:**
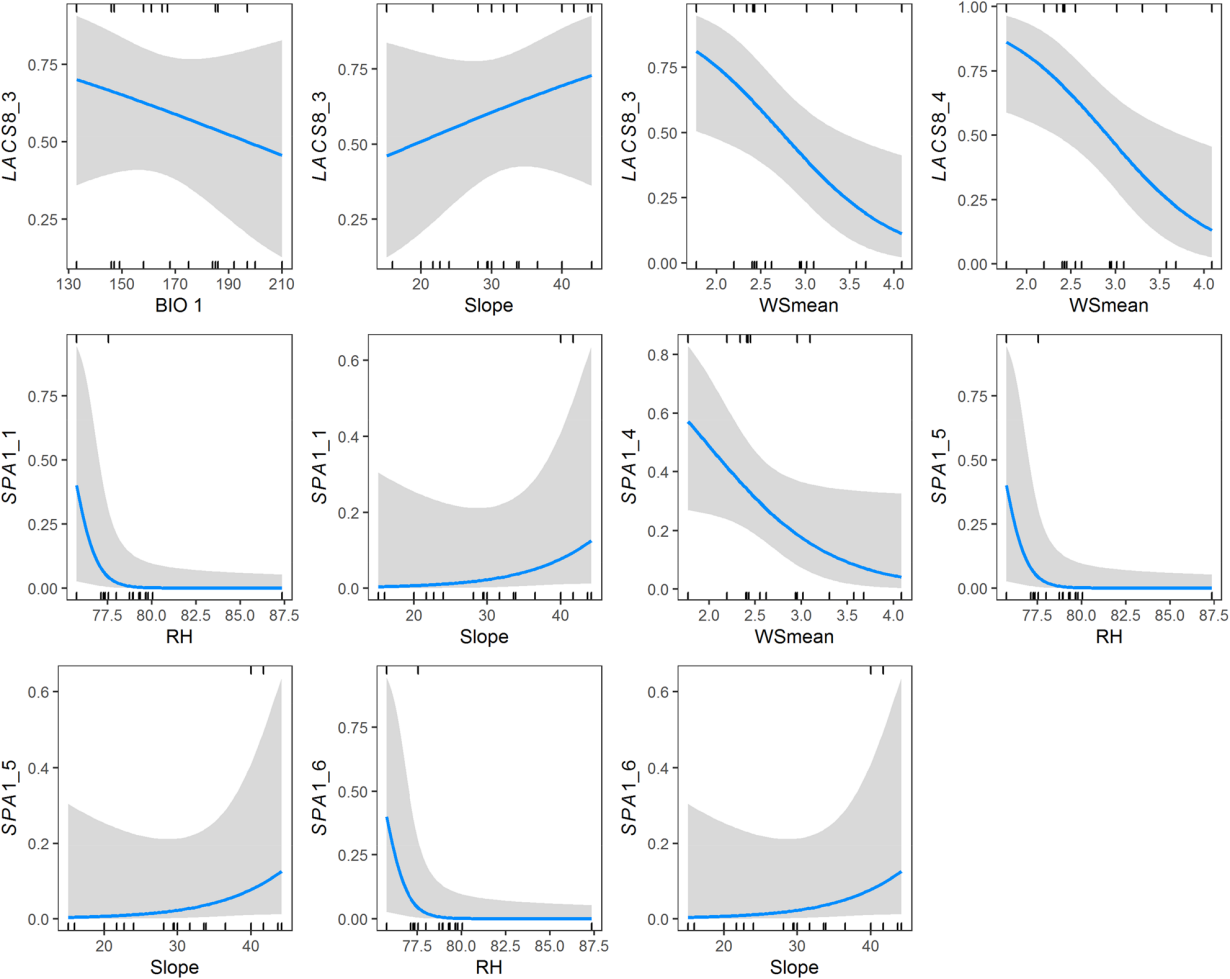
Logistic regression plots of the exon variants strongly correlated with environmental variables identified by both generalized linear and generalized linear mixed effect models presented in Table 5. Values of the y-axis represent the predicted probabilities of rare alleles of exon variants in *LACS8* and *SPA1* genes and numbers of the x-axis represent the values of environmental variables.

Seven out of the 31 exon variant alleles, including synonymous and nonsynonymous variations, were found to be strongly correlated with various combinations of environmental variables revealed either by generalized linear model (GLM) or generalized linear mixed effect model (GLMM), or by both GLM and GLMM (Table 5). The frequent to rare allele mutations in nonsynonymous substitution, including **G**CT → **T**CT in *LACS8_4*, C**G**A → C**A**A in *PCFS4_1*, C**A**C → C**G**C in *SPA1_1*, and A**A**T → A**G**T in *SPA1_6*, were found to be strongly associated either positively or negatively with various environmental variables (Table 5). Three synonymous variants, including GC**T** → GC**C** in *LACS8_3*, CT**C** → CT**G** in *SPA1_4*, and GT**A** → GT**C** in *SPA1_5*, were also found to be highly associated with environmental variables. Additionally, frequent alleles of these seven exon variants were found to be fixed in many populations across geographic regions (Fig. 2). For those exon variant alleles strongly correlated with environmental variables found by both GLM and GLMM, logistic regression plots were depicted (Fig. 3).

**Table 5:** Exon variable alleles strongly correlated with environmental variables based on generalized linear model (GLM) and generalized linear mixed-effects model (GLMM).

Exon variation	Frequent to rare allele change	Associated environmental variables	GLM		GLMM	
			*Z*	Estimate	*Z*	Estimate
*LACS8_*3	GC**T** → GC**C**^70^ (Ala → Ala) (S)	BIO1	− 2.661	− 0.037*^,^**^,^***	− 2.662	− 0.037*^,^**
		BIO7	− 2.449	− 0.071*^,^**		
		Slope	2.37	0.089*	2.284	0.092*
		WSmean	− 2.305	− 1.427*^,^**	− 2.31	− 1.513*
*LACS8*_4	**G**CT → **T**CT^122^ (Ala → Ser) (N_s_)	BIO1	− 2.063	− 0.026*		
		BIO7	− 2.826	− 0.087*^,^**^,^***		
		WSmean	− 2.085	− 1.184*	− 2.047	− 1.314*
*PCFS4*_1	C**G**A → C**A**A^134^ (Arg → Gln) (N_s_)	EVI	− 2.692	− 20.846*^,^**^,^***		
		NDVI	− 1.631	− 111.91*^,^**^,^***		
		RH			− 41.34	− 0.171*^,^**^,^***
*SPA1*_1	C**A**C → C**G**C^98^ (His → Arg) (N_s_)	Aspect			− 3.116	− 0.009*^,^**^,^***
		BIO7	− 2.541	− 0.208*^,^**^,^***		
		RH	− 2.903	− 2.095*^,^**^,^***	− 2.847	− 2.095*^,^**^,^***
		Slope	2.395	0.257*^,^**^,^***	2.172	0.269*
		WSmean	− 2.131	− 3.308*^,^**		
*SPA1*_4	CT**C** → CT**G**^104^ (Leu → Leu) (S)	WSmean	− 1.971	− 1.488*	− 1.97	− 1.488*
*SPA1*_5	GT**A** → GT**C**^113^ (Val → Val) (S)	Aspect			− 3.116	− 0.009*^,^**^,^***
		BIO7	− 2.541	− 0.208*^,^**^,^***		
		RH	− 2.903	− 2.095*^,^**^,^***	− 2.847	− 2.095*^,^**^,^***
		Slope	2.395	0.257*^,^**^,^***	2.172	0.269*
		WSmean	− 2.131	− 3.308*^,^**		
*SPA1*_6	A**A**T → A**G**T^115^ (Asn → Ser) (N_s_)	Aspect			− 3.116	− 0.009*^,^**^,^***
		BIO7	− 2.541	− 0.208*^,^**^,^***		
		RH	− 2.903	− 2.095*^,^**^,^***	− 2.847	− 2.095*^,^**^,^***
		Slope	2.395	0.257*^,^**^,^***	2.172	0.269*
		WSmean	− 2.131	− 3.308*^,^**		

*BIO1* annual mean temperature,* BIO7* temperature annual range,* EVI* enhanced vegetation index,* NDVI* normalized difference vegetation index,* RH* relative humidity,* WSmean* mean wind speed.

The superscript numbers on the second column represent amino acid position of the respective protein in *Rhododendron catawbiense.*

*S* synonymous substitution,* N*_s_ nonsynonymous substation.

*Values do not bracket zero in 95% confidence intervals.

**Values do not bracket zero in 99% confidence intervals.

***Values do not bracket zero in 99.5% confidence intervals.

Exon variable sites were coded as allelic presence ("1") and absence ("0") of the rare alleles and implemented in a generalized linear model (GLM) and a generalized linear mixed effect model (GLMM) as response variables to assess the correlations of exon variant alleles with environmental variables, with binomially distributed residuals.

The superscript numbers represent aligned exon sites for the nucleotide substitutions.

## Discussion

### Demographic history, genetic structure, and genetic diversity

Historical and contemporary demographic events played important roles in shaping the genetic structure of natural populations of species and traces in patterns of genetic diversity can be used to reveal population demographic history^41,42^. Climatic oscillations during the Pleistocene have been widely recognized as the main historical factor shaping current population genetic structure and distributions of species or populations^42^. Limited gene flow between geographic regions resulted from bottleneck events in the Holocene approximately 9168–13,092 years ago was revealed based on EST-SSRs in *R. oldhamii*^4^. This study inferred that *R. oldhamii* has experienced a process of transition from historical connectivity toward contemporary regional isolation. In the present study, we found no consistent evidence of spatial expansion based on Tajima’s *D* and Fu and Li’s D* and F* using DNA sequences of 12 nuclear gene loci (Table 2). However, spatial expansion cannot be rejected because significant small *R*_2_ values were found using the total aligned intron sequences of the pooled samples and the aligned intron sequences of the pooled samples of five of the 12 genes examined (Table 2). The discrepancy of the estimates of neutrality test statistics for different genes can be caused by a combination of factors, such as selection, demographic history, and differences in mutation rate^43,44^. Estimation of Tajima’s *D* and Fu and Li’s D* and F* was known to be influenced either by population reduction, population subdivision, a recent bottleneck, or migration which resulted in secondary contact among previously differentiated lineages^44–46^. Additionally, the power of statistical tests using Tajima’s *D* and Fu and Li’s D* and F* may be weak for small sample size, but *R*_2_ statistic is superior for small sample size^47^. Nonetheless, a coherent pattern of spatial expansion was also suggested by the SSD statistic considering population subdivision^43,44,48^ using the aligned intron sequences of the pooled samples of the 12 individual genes and the total aligned intron sequences of the pooled and the regional samples (Table 2).

Since nuclear DNA is the fastest evolving among the three genomes plants harbored^49^ and nuclear intron sequences evolving faster than coding sequences^50^, the nuclear intron sequences can reveal far-distant past demographic history in contrast to EST-SSRs located within protein-coding sequences. The estimation using formula *t* = *τ*/2*μk* suggests that the date of spatial expansion in *R. oldhamii* occurred during the late Pleistocene beginning approximately 68,784–119,685 years ago (Supplementary Table S6). In conjunction with the results of Hsieh et al.^4^, *R. oldhamii* could have experienced spatial expansion in the late Pleistocene followed by bottleneck events occurred in the Holocene. Historical spatial expansion might have resulted in the lack of clear genetic distinction among populations and the extremely low across population differentiation (*F*_ST_ = -0.0078, *P* = 0.331), due to the retention of ancestral polymorphisms, based on nuclear DNA intron sequence data in the present study. Hence, no clear genetic structuring was also observed based on DAPC (Supplementary Fig. S1). However, significant regional differentiation (*F*_ST_ = 0.074, *P* = 0.001) was observed which is consistent with the regional population divergence inferred based on EST-SSR and AFLP^4,5^.

Higher or comparable level of nucleotide diversity (*π* = 0.00659) was found based on the total aligned intron sequences of the 12 nuclear genes examined (Table 2) compared to the level of nucleotide diversity of other species endemic to Taiwan, such as *Cinnamomum kanehirae* (chalcone synthase: *π* = 0.00716 and leafy gene: *π* = 0.00479)^3^ and *Quercus glauca* (glyceraldehyde-3-phosphate dehydrogenase: *π* = 0.0050)^51^. Moreover, the level of nucleotide diversity of *R. oldhamii* was found to be higher than the level of nucleotide diversity (*π* = 0.00134) of a mainland *Rhododendron* species, *R. delavayi*, based on sequences of a major RNA Polymerase II subunit^52^ and the nucleotide diversity (*π* = 0.0039) based on eight nuclear loci of a *Rhododendron* species, *R. weyrichii*, distributed in Japan and South Korea^53^. Nonetheless, the comparisons may not be appropriate because DNA sequences used in the calculation of nucleotide diversity were derived from different genes. Although there are no nucleotide diversity estimates based on DNA sequences available for comparison, the level of genetic diversity can be compared between congeneric species occurring in Taiwan based on EST-SSRs genotyped using the same set of amplification primer pairs. Comparable levels of *R. oldhamii* EST-SSR genetic diversity across populations (average *H*_E_ = 0.284)^4^ were found when compared with other *Rhododendron* species belonging to the same subgenus *Tsutsusi* occurring in Taiwan (average *H*_E_ = 0.293)^54^.

Forests in Taiwan were known to have a 1,500–1,600 m upward migration since the last glacial maximum^55^. In parallel with rising temperatures due to climate changes, upper altitudinal limits of mountain plants have been found to increase at a rate of ca. 3.6 m per year during the past century on the subtropical island of Taiwan and survival of plant species has been greatly affected^56^. It is probable that suitable ecological niches for the warmth-loving *R. oldhamii* living in the humid understory of forests could be reduced because of range retractions^57^. However, the response to forest fragmentation may differ in congeneric species adapted to different habitat types^58^. *R. oldhamii* harbors lower level of EST-SSR genetic diversity compared with other endemic species of the *R. pseudochrysanthum* complex belonging to the subgenus *Hymenanthesis* (average *H*_E_ = 0.424)^59^. The level of EST-SSR genetic diversity may not only reflect the outcome of a long evolutionary history, but also influenced by recent demographic events^2,3,42,60^. The lower level of EST-SSR genetic diversity in *R. oldhamii* compared with species of the *R. pseudochrysanthum* complex might have been resulted from inbreeding (Table 1) due to bottlenecks caused by habitat fragmentation in the recent past^4,61^ in contrast to congeneric species of the *R. pseudochrysanthum* complex that experienced no bottlenecks^59^.

### Environmental variables strongly associated with synonymous and nonsynonymous variants of *LACS8* and *SPA1* nuclear genes

Elucidating the potential role of natural genetic variation in association with ecological factors has been important in evolutionary biology^62^. Environmental heterogeneity due to landscape complexity can have great influence on distribution of mountain species^63^, and rugged topography and steep altitudinal environmental gradients, ranging from deep valleys to 3,000 m peaks, are common in the mountainous regions of Taiwan^64^^,^^65^. *R. oldhamii* distributed in an elevational range from 136 to 1868 m spanning wide environmental gradients (Supplementary Table S9) that may have played important roles in shaping population adaptive evolution. In the present study, we used logistic regression approaches, including GLM and GLMM, to test for the most influential environmental variables strongly correlated with synonymous and nonsynonymous variants in *LACS8* and *SPA1* genes (Table 5 and Fig. 3).

Our results found contrasting trends of changes in environmental variables between slope and other environmental variables associated with changes in the predicted probabilities of exon rare variant alleles of *LACS8* and *SPA1* (Fig. 3). The frequent alleles of the exon variants of these two genes were highly associated with higher values of environmental variables including BIO1, WSmean, and RH, but strongly associated with lower slope values (Table 5, Fig. 3). These results suggest that there were exon variants, particularly the frequent synonymous variants, played important roles in adapting to higher values of BIO1, WSmean, and RH, and individuals possessed these exon variations inhabiting the flatter, with smaller environmental variance, rather than the steep mountain slopes. Evidence of temperature plays an important role as an ecological driver either for adaptive genetic or epigenetic variation has been widely detected for diverse plant species distributed in different parts of the world^5,66–70^. Slope as a topological factor can act as a heat source in the day time and the surface temperature become warmer than the free atmosphere^71,72^. Warmer ambient temperature can hold moisture in the air resulting in higher relative humidity. Mean wind speed influenced by monsoon could be an important factor determining vegetation occurring in Taiwan apart from climatic factors among different altitudinal and geographic regions^73^. Our results in the present study found that environmental factors such as BIO1, BIO7, WSmean, RH, and slope strongly associated with coding sequence variation in natural populations of *R. oldhamii* suggestive of local adaptation in consistence with the findings of previous studies^4,5^.

*LACS8* is one member of long-chain acyl-coenzyme A synthetase gene family found in *Arabidopsis* and *LACS* mutants were found to have a damping effect on endoplasmic reticulum to plastid lipid trafficking causing lethality^74^. *SPA* encoding suppressor of phyA-105 proteins which are involved in regulating light dependent developmental processes, including photoperiodic flowering^75^. It is likely that BIO1, WSmean, and slope played important roles in driving adaptive variation of *LACS8* gene. The protein encoded by *LACS8* was known to play an important role in signaling that governs biotic and abiotic stress responses, including temperature-induced stress that provokes changes in plasma membrane physico-chemical properties^76^. Moreover, adaptive variation in *SPA1* could be driven mainly by RH, WSmean, and slope, and might have been important to flowering response^75^ of *R. oldhamii* individuals grown in different geographic regions^34,35^.

### Synonymous substitutions at third codon positions may have been the targets favored by selection

Selection was demonstrated to play an important role in driving codon usage pattern^18,26,30^, albeit codon bias has been attributed mostly to neutral forces, such as mutational bias and GC bias conversion^30^. In flowering plants, relationship between GC content of coding and non-coding sequences are heterogeneous among genes^77^, and we found no significant positive correlations in the average GC content of coding sites and of third codon positions, respectively, with the average GC content of surrounding non-coding regions across all genes based on Spearman’s rank correlation test (*ρ* = − 0.434, *P* = 0.161; *ρ* = 0.147, *P* = 0.651, respectively). In addition, background compositional bias may also play an important role in the synonymous codon selection in a gene^30,78^. Although, intron length may have a positive relationship with the level of intron GC content^79^, we found no significant correlation between length of intron and its GC content of the 12 nuclear genes examined (Spearman’s *ρ* = 0.007, *P* = 0.991). Additionally, the average GC content of coding sites and of third codon positions were significantly different from the average GC content of surrounding non-coding regions (Table 3). Exon length may not be the influential factor causing the difference because no positive correlations in the average GC content of coding sites and of third codon positions, respectively, with exon length were found across all genes (Spearman’s *ρ* = 0.074, *P* = 0.820 and Spearman’s *ρ* = 0.056, *P* = 0.863, respectively). Nonetheless, selection might have played an important role in the levels of GC content at both synonymous and nonsynonymous substitution sites^13,14^.

*LACS8* and *SPA1* were the two genes that had lesser amounts of average GC content at third codon positions compared to that of surrounding non-coding regions (Table 3). Although the level of GC content at third codon positions was found to be positively correlated with the level of gene expression^29,30^, our results found that synonymous variants at third codon positions of *LACS8* and *SPA1,* including variants of T/C (*LACS8_3*) and A/C (*SPA1_5*), possessed lesser amounts of GC content compared to surrounding non-coding regions (Table 3). The frequent T and A alleles in these two genes, respectively, were found to be closely associated with environmental variables (Table 5). Additionally, fixation of these two synonymous frequent alleles in many populations across geographic regions was found (Fig. 2). Although only partial coding sequences were examined and optimal codons may be more frequently end in G or C in dicots^14,32,33^, our results suggest that optimal codons may not use G or C at third codon positions in *LACS8* and *SPA1* as expected, and they might have correlated with the optimal expression of these genes favored by selection^25,80,81^.

## Conclusions

Understanding the phylogeographic pattern of species and population adaptive evolution is important in evolutionary biology. In the present study, we haphazardly selected 12 nuclear loci for sequencing of natural population individuals and used in phylogeographic study and testing for adaptive evolution. The results of the present study in conjunction with the results of the previous study^4^ suggest that *R. oldhamii* experienced spatial expansion in the far-distant past during the late Pleistocene followed by the recent bottlenecks in the Holocene resulting in population differentiation at regional scale. Exon variation was found to be significantly explained by environmental variables. Environmental variables that might have invoked strong selection on the seven adaptive exon variants were BIO1, WSmean, RH, and slope. Our results found causal associations of *LACS8* and *SPA1* genes, including synonymous and nonsynonymous variations, with environments in *R. oldhamii*. Our study suggests that synonymous variants, particularly those codons end in either T or A rather than G or C as expected in dicots^32,33^, of nuclear genes may act as optimal codons with high frequency involved in adaptive divergence related to stress and flowering response of natural *R. oldhamii* populations located in different geographic regions.

## Methods

### Samples and nuclear loci

Previous studies demonstrated that *R. oldhamii* populations can be classified geographically into four and three regional groups based on genotypic data of EST-SSR^6^ and AFLP^7^, respectively. The four regional groups based on EST-SSR were north, central, south, and southeast groups^4^ (Table 1). The population genetic structuring of the 18 *R. oldhamii* populations analyzed based on EST-SSR and AFLP agreed with each other, except that the EST-SSR central group contains population WL (Table 1, Fig. 1), but population WL was clustered into the north group based on AFLP. We adopted the EST-SSR clustering for the present study. The number of samples collected for each geographic region ranged from 8 to 15 (north, *n* = 14; central, *n* = 8; south, *n* = 15; and southeast, *n* = 10). These samples were used for DNA extraction^82^ and in direct sequencing of polymerase chain reaction (PCR)-amplified DNA products of 12 nuclear loci (Supplementary Methods and Supplementary Table S1).

### PCR and sequencing

PCR primers for the 12 genes (Supplementary Methods and Supplementary Table S10) were designed using PRIMER 3 (https://bioinfo.ut.ee/primer3-0.4.0/) based on EST sequences of *R. catawbiense*^83^. PCR amplifications were performed in a PTC-100 DNA programmable thermal cycler (MJ Research, Watertown, MA, USA) and done by initial denaturation (98 °C, 3 min), 40 cycles of denaturation (98 °C, 1 min), annealing (53.5–61.9 °C, 1 min) (Supplementary Table S10) and extension (72 °C, 1 min), and final extension (72 °C, 5 min) in a total of 40 μL PCR buffer. The PCR buffer contains 40 ng template DNA, 1X Phusion HF buffer, 1.5 mM MgCl_2_, 0.2 mM deoxyribonucleotide triphosphate mix, 0.5 μM primer, and 2 U of Phusion Hot Start DNA polymerase (Finnzymes Oy, Espoo, Finland). The amplification products were electrophoresed on a 1% agarose gel and the corresponding bands of the 12 genes under study were purified with Viogene Gel Extraction Kit (Viogene, Taipei, Taiwan) and directly sequenced using an ABI 3730 DNA sequencer (Applied Biosystems, Foster City, CA). Heterozygous site resolution, haplotype phasing, and functional annotation (Supplementary Table S11) were described in Supplementary Methods.

### Sequence alignment, summary statistics, and neutrality tests

Sequence alignment was performed using the *msa* function of R msa package^84^ based on the ClustalW algorithm^85^ in the R environment^86^. Summary statistics including the indices of the average number of pairwise nucleotide differences per site (*π*)^87^, the average nucleotide diversity of segregating sites (*θ*_w_)^88^, and haplotype diversity (*H*_*d*_) were computed using DNASP v.6^89^. Neutrality test statistics including Tajima’s *D*^90^, Fu and Li’s D* and F*^91^, and *R*_2_^47^ were also estimated using DNASP and tested for deviation from neutral expectation using 10,000 coalescent simulations. Summary statistics were estimated based on the total aligned intron sequences of the population, regional and pooled samples, and computed for the aligned intron sequences of the pooled samples for each gene separately. Neutrality test statistics were computed based on the total aligned intron sequences of the regional samples and the pooled samples and the aligned intron sequences of the pooled samples for each locus individually. Significant negative values of *D*, D*, and F* and significant small positive values of *R*_2_ represent an excess of low frequency mutations, indicating unimodal mismatch distributions, representative of sudden expansion relative to a null model of demographic stability with multimodal mismatch distributions. The minimum number of *R*_m_ following the four-gamete test^92^ and the number of segregating sites were estimated for each gene based on the aligned intron sequences of the pooled samples using DNASP. A goodness-of-fit test based on the SSD statistic was calculated using ARLEQUIN v.3.5^93^ for the total aligned intron sequences of the regional and the pooled samples and the aligned intron sequences of the pooled samples of each gene separately considering population subdivision. A significant SSD value represents departure from the estimated demographic model of spatial expansion. In the goodness-of-fit test, though the error estimate is generally high, the time of spatial expansion was calculated using the formula *t* = *τ*/2*μk*, where *t* is the time since the expansion, *τ* is the estimated number of generations since the expansion, *μ* is the mutation rate per site per generation, and *k* is the sequence length. We adopted a generation time of 15 years^53,94,95^ and the mutation rate of 1.581 × 10^–9^ per site per year^53^ used in the study of population demographic history of *R. weyrichii*^53^, which is also belongs to the subgenus *Tsutsusi*, for calculation of the expansion time. Friedman test was used to assess the overall difference of nucleotide diversity (*π* and *θ*_w_) at the population and regional levels using the *friedman* function of R agricolae package^96^. In Freidman test, nuclear locus was used as a blocking effect. Pairwise population and regional comparisons were performed and *P* values adjusted using Fisher’s least significant difference.

### The average GC content

The GC content of 94 sequences derived from 47 individuals at coding sites, at third codon positions, and in surrounding non-coding regions of the 12 genes were calculated using CodonW (https://codonw.sourceforge.net//culong.html). Differences in the mean GC content of coding sites and of third codon positions compared with the average GC content of surrounding non-coding regions of each gene were assessed using paired Wilcoxon test (the R *wilcox.test* function of R).

### Inbreeding coefficient and genetic structure

The total aligned intron sequences of the samples of each population were used in estimating 95% confidence intervals (CIs) of *F*_IS_ using the *boot.ppfis* function of R hierfstat package^97^ with 999 bootstrap resampling, and means and *P* values were calculated based on *Z* distribution. Across region/population *F*_ST_ and pairwise *F*_ST_ comparisons were estimated based on the aligned intron sequences using the *popStructTest* function of R package strataG^98^ based on 999 permutations. Population structure was evaluated with DAPC^99^ based on the total aligned intron sequences of the pooled samples using the *find.clusters* and *dapc* functions of R adegenet package^100^.

### Environmental variables

Environmental variables with variance inflation factor (VIF) > 5 and highly correlated with other variables (|r|> 0.8) were removed (Supplementary Methods and Supplementary Table S12). Eight environmental variables: BIO1, BIO7, aspect, slope, EVI, NDVI, RH, and WSmean were retained as explanatory variables (Supplementary Table S9).

PERMANOVA was used to assess environmental heterogeneity, based on the eight retained environmental variables, among populations and among regions using the *adonis* function of R package vegan^101^. In PERMANOVA, environmental Euclidean distance matrix was used as response variable to test the differences among populations and among regions. Significance was determined with 999 permutations. The *pairwise.perm.manova* function of R package RVAideMemoire^102^ was used in pairwise comparisons, and significance determined by 999 permutations and an FDR of 5%.

### Associations of exon variant alleles with environmental variables

Exon variable sites were coded as allelic presence ("1") and absence ("0") of the rare alleles and implemented in a GLM and a GLMM as response variables to assess the correlations of exon variant alleles with environmental variables, with binomially distributed residuals, and significance assessed with 95%, 99%, and 99.5% CIs. GLMs were performed using the R *glm* function. In GLMMs, environmental variables were used as fixed effects and geographic region as a random effect and analyzed using the *glmer* function of R lme4 package^103^. Exon variant alleles found to be significantly correlated with environmental variables detected by both GLM and GLMM were used in the visualization of the probability estimates against the associated environmental gradients using the *visreg* function of R visreg package^104^.

### Disentangling the effects of environment and geography explaining exon variation

The frequencies of the frequent alleles of exon variants were used in a variation partitioning analysis to disentangle the effects of environment and geography explaining exon variation. The *varpart* and *anova.cca* functions of R package vegan were used, respectively, for variation partitioning and testing for significance with 999 permutations. Exon variation was partitioned into four fractions explained by pure environmental variables (fraction [a]), geographically-structured environmental variables (fraction [b]), pure geographic variables (fraction [c]), and residual effects (fraction [d])^105,106^, based on adjusted *R*^2^ values^107^. Sample site coordinates were used as geographic variables in variation partitioning.

## Supplementary information

Supplementary information.
