# Supplementary material for: Demographic history and adaptive synonymous and nonsynonymous variants of nuclear genes in Rhododendron oldhamii (Ericaceae)
Source: Sci Rep. 2020 Oct 7;10:16658. doi: 10.1038/s41598-020-73748-z (PMC7542430; doi:10.1038/s41598-020-73748-z)

**SUPPLEMENTARY INFORMATION**

**"Demographic history and adaptive synonymous and nonsynonymous variants of nuclear genes in *Rhododendron oldhamii* (Ericaceae)"**

Yi-Chiang Hsieh^1^, Chung-Te Chang^2^, Jeng-Der Chung^3^ and Shih-Ying Hwang^1*^

^1^ School of Life Science, National Taiwan Normal University, 88 Tingchow Road, Section 4, Taipei 11677, Taiwan,

^2^ Department of Life Science, Tunghai University, 1727 Taiwan Boulevard, Section 4, Taichung 40704, Taiwan,

^3^ Division of Silviculture, Taiwan Forestry Research Institute, 53 Nanhai Road, Taipei 10066, Taiwan

*Corresponding author: hsy9347@ntnu.edu.tw

School of Life Science, National Taiwan Normal University, 88 Tingchow Road, Section 4, Taipei 11677, Taiwan,

Tel: +886-2-77346262

Fax: +886-2-29312904

**Supplementary Methods:**

**Nuclear loci and haplotype determination.** Twelve nuclear loci were haphazardly selected including altered meristem program 1 (*AMP1*), MYB domain protein 33 (*ATMYB33*), constitutive photomorphogenic dwarf (*CPD*), glyceraldehyde-3-phosphate dehydrogenase C subunit 1 (*GAPC1*), glycine rich protein 7 (*GRP7*), uroporphyrinogen decarboxylase 2 (*HEME2*), long-chain acyl-COA synthase 8 (*LACS8*), photosystem 1 light harvesting complex gene 1 (*LHCA1*), polyadenylation and cleavage factor-similar protein 4 (*PCFS4*), peroxisomal NAD-malate dehydrogenase 2 (*PMDH2*), suppressor of phyA-105 (*SPA1*), and protein translation factor SUI1 homolog (*SUI1*). The proteins of these genes have been found to play roles involving in, e.g., signaling, growth, development, and stress response.

ContigExpress software (Vector NTI suite v.9, Invitrogen, Carisbad, CA, USA) was used to call heterozygous sites based on secondary peak height with a threshold of 50% in the chromatogram. Indelligent software v.1.2^1^ was used to resolve heterozygous insertion-deletion (indel) events that lead to overlapping chromatograms within sequences. Sequences were trimmed and assembled manually using BioEdit^2^ after visual examination of the base calls. The most probable haplotype pair for all individuals was determined using PHASE^3,4^ by assessing the posterior probability of each possible haplotype with 10,000 iterations of the Bayesian Markov chain Monte Carlo (MCMC) chain, sampling every 10 iterations with a burn-in of 100 iterations. The accepted haplotypes inferred by PHASE were those with a posterior probability of ≥ 0.9 and most of them had a posterior probability equals to 1^5^. Functional annotation and exon and intron positions of the corresponding genes were blasted against available sequences from GenBank data base using BALSTX (Supplementary Table S11). The DNA sequences of haplotypes obtained from the 12 studied genes have been deposited in GenBank (Supplementary Table S1).

**Environmental variables.** We obtained 19 bioclimate, two topographic, and 11 ecological variables as environmental variables for sample sites. We downloaded bioclimate variables from the WorldClim v.1.4 (http://www.worldclim.org/) at 30-sec spatial resolution (~ 1 km)^6^. Two topographic variables (aspect and slope) at 30-m resolution were acquired from Global Digital Elevation Map (http://lpdaac.usgs.gov). Moderate resolution imaging spectroradiometer (MODIS) datasets during 2001‒2013 (acquired from Land Process Distributed Active Archive Center, LPDAAC, http://lpdaac.usgs.gov) were used to obtain ecological variables including normalized difference vegetation index (NDVI) and enhanced vegetation index (EVI) derived from dataset MOD13A2 (1 km resolution), and leaf area index (LAI) and fraction of absorbed photosynthetically active radiation (fPAR) derived from MOD15A2 dataset (500 m resolution), and the annual total potential evapotranspiration (PET) derived from MOD16A3 dataset (500 m resolution). We computed the monthly mean values using a maximum-value composite procedure^7^.

Moreover, ecological variables including relative humidity (RH), cloud cover (CLO), time of sunshine (SunH), number of rainfall days per year (RainD), and mean wind speed (WSmean), at spatial resolution of 1 km, were obtained from the Data Bank for Atmospheric & Hydrologic Research (https://dbahr.pccu.edu.tw/, recorded in 1990–2013), and monthly mean values calculated using a universal spherical model of the Kriging method in ArcGIS^8^. Soil pH values of sample sites based on an island-wide soil investigation (*n* = 1150) conducted in 1969‒1986 ^9^ were also calculated using ArcGIS. Annual moisture index^10^ was computed from annual precipitation and annual potential evapotranspiration (derived from annual mean temperature).

Correlations between environmental variables were calculated using the *cor* function of R^11^. Variance inflation factor (VIF) was calculated using the *vif* function of R package usdm^12^. Environmental variables with VIF > 5 and highly correlated with other variables (|r| > 0.8) were removed (Supplementary Table S12).

**Supplementary references:**

1. Dmitriev, D. A. & Rakitov, R. A. Decoding of superimposed traces produced by direct sequencing of heterozygous indels. *PLoS Comput. Biol.* **4,** e1000113, https://doi.org/10.1371/journal.pcbi.1000113 (2008).

2. Hall, T. BioEdit: An important software for molecular biology. *GERF Bull. Biosci.* **2,** 60–61 (2011).

3. Stephens, M., Smith, N. J. & Donnelly, P. A new statistical method for haplotype reconstruction from population data. *Am. J. Hum. Genet.* **68,** 978–989, https://doi.org/10.1086/319501 (2001).

4. Stephens, M. & Donnelly, P. A comparison of bayesian methods for haplotype reconstruction from population genotype data. *Am. J. Hum. Genet.* **73,** 1162–1169, https://doi.org/10.1086/379378 (2003).

5. Garrick, R. C., Sunnucks, P. & Dyer, R. J. Nuclear gene phylogeography using PHASE: dealing with unresolved genotypes, lost alleles, and systematic bias in parameter estimation. *BMC Evol. Biol.* **10,** 118, https://doi.org/10.1186/1471-2148-10-118 (2010).

6. Hijmans, R. J, Cameron, S. E., Parra, J. L., Jones, P. G. & Jarvis, A. Very high resolution interpolated climate surfaces for global land areas. *Int. J. Climatol.* **25,** 1965–1978, https://doi.org/10.1002/joc.1276 (2005).

7. Huete, A. R., Didan, K., Miura, T., Rodriguez, E. P., Gao, X. & Ferreira, L. G. Overview of the radiometric and biophysical performance of the MODIS vegetation indices. *Remote Sens. Environ.* **83,** 195–213, https://doi.org/10.1016/S0034-4257(02)00096-2 (2002).

8. Chang, C.-T., Wang, S.-F., Vadeboncoeur, M. A. & Lin, T.-C. Relating vegetation dynamics to temperature and precipitation at monthly and annual timescales in Taiwan using MODIS vegetation indices. *Int. J. Remote Sens.* **35,** 598–620, https://doi.org/10.1080/01431161.2013.871593 (2014).

9. Chang, C.-T., Lin, T.-C. & Lin, N.-H. Estimating the critical load and the environmental and economic impact of acid deposition in Taiwan. *J. Geogr. Sci.* **56,** 39–58 (2009)

10. Thornthwaite, C. W. An approach toward a rational classification of climate. *Geogr. Rev.* **38,** 55–94, (1948).

11. R Core Team. R: A Language and Environment for Statistical Computing. <https://www.R-project.org/> (R Foundation for Statistical Computing, Vienna, Austria, 2018).

12. Naimi B, Hamm NAS, Groen TA, Skidmore AK, Toxopeus AG. 2014. Where is positional uncertainty a problem for species distribution modelling? *Ecography* **37:** 191–203.

**Supplementary Table S1.** GenBank accession numbers for the haplotypes of the 12 nuclear genes examined in *Rhododendron oldhamii*.

| Genes | Abbreviation | Accession number |
| --- | --- | --- |
| Glutamate carboxypeptidase | *AMP1* | MN018125–MN018153 |
| Myb domain protein 33 | *ATMYB33* | MN027069–MN027095 |
| Cytochrome P450 90A1 | *CPD* | MN018105–MN018124 |
| Glyceraldehyde-3-phosphate dehydrogenase C subunit 1 | *GAPC1* | MN018074–MN018104 |
| Glycine-rich RNA-binding protein 7 | *GRP7* | MN018050–MN018073 |
| Uroporphyrinogen decarboxylase | *HEME2* | MN018016–MN018049 |
| Long chain acyl-coa synthetase 8 | *LACS8* | MN017994–MN018015 |
| Chlorophyll a-b binding protein 6 | *LHCA1* | MN026925–MN026941 |
| PCF11P-similar protein 4 | *PCFS4* | MN026942–MN026972 |
| Peroxisomal NAD-malate dehydrogenase 2 | *PMDH2* | MN026973–MN027011 |
| Protein SUPPRESSOR OF PHYA-105 | *SPA1* | MN027012–MN027033 |
| Protein translation factor SUI1 | *SUI1* | MN027034–MN027068 |

**Supplementary Table S2.** The length and positions of exon and intron within each of the 12 loci in *Rhododendron oldhamii*.

| Locus | L_total_ | L_exon_ | Number of codon | L_intron_ |
| --- | --- | --- | --- | --- |
| *AMP1* | 804 | 318 (338-480, 630-804) | 106 | 486 (1-337, 481-629) |
| *ATMYB33* | 776 | 81 (1-38, 734-776) | 27 | 695 (39-733) |
| *CPD* | 880 | 153 (1-23, 132-210, 830-880) | 51 | 727 (24-131, 211-829) |
| *GAPC1* | 509 | 153 (1-48, 240-323, 489-509) | 51 | 356 (49-239, 324-488) |
| *GRP7* | 895 | 36 (1-21, 881-895) | 12 | 859 (22-880) |
| *HEME2* | 804 | 171 (1-39, 427-539, 786-804) | 57 | 633 (40-426, 540-785) |
| *LACS8* | 704 | 372 (1-64, 177-307, 388-563, 704-704) | 124 | 332 (65-176, 308-387, 564-703) |
| *LHCA1* | 616 | 336 (134-423, 571-616) | 112 | 280 (1-133, 424-570) |
| *PCFS4* | 908 | 78 (1-37, 868-908) | 26 | 830 (38-867) |
| *PMDH2* | 643 | 105 (1-23, 154-235) | 35 | 538 (24-153, 236-643) |
| *SPA1* | 620 | 117 (1-60, 564-620) | 39 | 503 (61-563) |
| *SUI1* | 712 | 162 (1-152, 703-712) | 54 | 550 (153-702) |

L_total_, the total length of the sequence; L_intron_, the length of intron; L_exon_, the length of exon.

The length of exon and intron are shown in base pair. Positions of exon and intron within each of the 12 loci are shown in parentheses.

**Supplementary Table S3.** Nucleotide diversity (*π*) for the 18 *Rhododendron oldhamii* populations.

| Population | *AMP1* | *ATMYB33* | *CPD* | *GAPC1* | *GRP7* | *HEME2* | *LACS8* | *LHCA1* | *PCFS4* | *PMDH2* | *SPA1* | *SUI1* |
| --- | --- | --- | --- | --- | --- | --- | --- | --- | --- | --- | --- | --- |
| BL | 0.00406 | 0.0015 | 0.00167 | 0.01959 | 0.00116 | 0.01933 | 0.00502 | 0.0009 | 0.00194 | 0.01018 | 0.00135 | 0.01169 |
| EGS | 0.00413 | 0 | 0.00138 | 0.03134 | 0.00466 | 0.03165 | 0 | 0.0036 | 0.00483 | 0 | 0.00797 | 0.01097 |
| HYS | 0.00372 | 0.00527 | 0.00153 | 0.02025 | 0.00088 | 0.0119 | 0.00221 | 0.00199 | 0.00426 | 0.01154 | 0.00549 | 0.00276 |
| STS | 0 | 0.00145 | 0 | 0.01425 | 0 | 0.03006 | 0 | 0 | 0.00121 | 0.01313 | 0.00596 | 0.00366 |
| TGK | 0 | 0.00145 | 0 | 0.02849 | 0.00466 | 0.00158 | 0.00311 | 0.00358 | 0 | 0.01126 | 0 | 0.00183 |
| TKL | 0 | 0 | 0 | 0 | 0 | 0 | 0 | 0 | 0.00241 | 0.01301 | 0.00199 | 0.03108 |
| WLJ | 0 | 0 | 0 | 0.0069 | 0.00116 | 0 | 0.00311 | 0 | 0.00121 | 0.01313 | 0.00797 | 0.02742 |
| WL | 0.04245 | 0.00154 | 0.00107 | 0 | 0.00115 | 0.01718 | 0.00333 | 0.00161 | 0.00293 | 0.0096 | 0.00105 | 0.01303 |
| CH | 0 | 0 | 0 | 0 | 0 | 0 | 0.00625 | 0 | 0 | 0.00956 | 0.00398 | 0.02559 |
| CJ | 0.00207 | 0 | 0 | 0 | 0 | 0.00158 | 0.00621 | 0 | 0.00483 | 0.00558 | 0 | 0 |
| CY | 0.00262 | 0.00376 | 0.00073 | 0.01829 | 0.00116 | 0.02184 | 0.00333 | 0.00239 | 0.00531 | 0.00932 | 0.00716 | 0.01038 |
| HS | 0.0031 | 0.00241 | 0 | 0.01952 | 0.00175 | 0.01846 | 0.00313 | 0.00657 | 0.00241 | 0.01338 | 0.00133 | 0.01554 |
| LLK | 0.00162 | 0.00264 | 0.00093 | 0.01265 | 0.00108 | 0.01712 | 0.00346 | 0 | 0.00517 | 0.01336 | 0.00156 | 0.01583 |
| LS | 0.0031 | 0 | 0.00161 | 0.02818 | 0 | 0.01635 | 0.00417 | 0.00358 | 0.00483 | 0.01474 | 0.0053 | 0.01798 |
| RL | 0.00413 | 0.00289 | 0 | 0 | 0.00233 | 0 | 0.00625 | 0 | 0.00241 | 0.01145 | 0 | 0 |
| WS | 0.0062 | 0.00289 | 0 | 0.03714 | 0.00233 | 0.03165 | 0 | 0.00358 | 0.00242 | 0.01336 | 0.00199 | 0 |
| WR | 0.0017 | 0.00215 | 0.00101 | 0.00101 | 0.00041 | 0.0129 | 0.00896 | 0.00581 | 0.00407 | 0.00595 | 0.00212 | 0.01857 |
| YP | 0.00331 | 0.00081 | 0.00129 | 0.01352 | 0.00106 | 0.01382 | 0.00514 | 0.01466 | 0.00241 | 0.00575 | 0.00565 | 0.01446 |

*π*, *the average number of pairwise nucleotide differences per site.*

**Supplementary Table S4.** Nucleotide diversity (*θ_w_*) for the 18 *Rhododendron oldhamii* populations.

| Population | *AMP1* | *ATMYB33* | *CPD* | *GAPC1* | *GRP7* | *HEME2* | *LACS8* | *LHCA1* | *PCFS4* | *PMDH2* | *SPA1* | *SUI1* |
| --- | --- | --- | --- | --- | --- | --- | --- | --- | --- | --- | --- | --- |
| BL | 0.00478 | 0.00167 | 0.00159 | 0.01433 | 0.0018 | 0.01404 | 0.00482 | 0.00138 | 0.00233 | 0.01013 | 0.00153 | 0.0134 |
| EGS | 0.00413 | 0 | 0.00138 | 0.03134 | 0.00466 | 0.03165 | 0 | 0.0036 | 0.00483 | 0 | 0.00797 | 0.01097 |
| HYS | 0.00292 | 0.00512 | 0.00195 | 0.01313 | 0.00124 | 0.01232 | 0.0022 | 0.00253 | 0.00512 | 0.01088 | 0.00493 | 0.00388 |
| STS | 0 | 0.00145 | 0 | 0.01425 | 0 | 0.03006 | 0 | 0 | 0.00121 | 0.01313 | 0.00596 | 0.00366 |
| TGK | 0 | 0.00145 | 0 | 0.02849 | 0.00466 | 0.00158 | 0.00311 | 0.00358 | 0 | 0.01126 | 0 | 0.00183 |
| TKL | 0 | 0 | 0 | 0 | 0 | 0 | 0 | 0 | 0.00241 | 0.01301 | 0.00199 | 0.03108 |
| WLJ | 0 | 0 | 0 | 0.01378 | 0.00116 | 0 | 0.00311 | 0 | 0.00121 | 0.01313 | 0.00797 | 0.02742 |
| WL | 0.05458 | 0.00218 | 0.00166 | 0 | 0.00246 | 0.01097 | 0.00188 | 0.00216 | 0.00291 | 0.00983 | 0.0012 | 0.01047 |
| CH | 0 | 0 | 0 | 0 | 0 | 0 | 0.00625 | 0 | 0 | 0.00956 | 0.00398 | 0.02559 |
| CJ | 0.00207 | 0 | 0 | 0 | 0 | 0.00158 | 0.00621 | 0 | 0.00483 | 0.00558 | 0 | 0 |
| CY | 0.00271 | 0.00444 | 0.0006 | 0.01502 | 0.00153 | 0.01663 | 0.00274 | 0.00314 | 0.00635 | 0.00923 | 0.00522 | 0.01364 |
| HS | 0.00338 | 0.00237 | 0 | 0.0187 | 0.0019 | 0.01985 | 0.00341 | 0.00587 | 0.00197 | 0.01356 | 0.00108 | 0.01695 |
| LLK | 0.00159 | 0.00336 | 0.00106 | 0.01322 | 0.00135 | 0.0122 | 0.00362 | 0 | 0.00419 | 0.01398 | 0.00153 | 0.01342 |
| LS | 0.00338 | 0 | 0.0015 | 0.02657 | 0 | 0.01726 | 0.00341 | 0.00391 | 0.00526 | 0.01469 | 0.00434 | 0.01795 |
| RL | 0.00413 | 0.00289 | 0 | 0 | 0.00233 | 0 | 0.00625 | 0 | 0.00241 | 0.01145 | 0 | 0 |
| WS | 0.0062 | 0.00289 | 0 | 0.03714 | 0.00233 | 0.03165 | 0 | 0.00358 | 0.00242 | 0.01336 | 0.00199 | 0 |
| WR | 0.00146 | 0.00205 | 0.00097 | 0.00101 | 0.00041 | 0.01454 | 0.00773 | 0.00633 | 0.00597 | 0.00723 | 0.00281 | 0.01292 |
| YP | 0.00365 | 0.00102 | 0.00146 | 0.01313 | 0.00123 | 0.01454 | 0.00552 | 0.01014 | 0.00301 | 0.00597 | 0.00562 | 0.01163 |

*θ_w_*, *the average nucleotide diversity of segregating site*.

**Supplementary Table S5.** Results of post-hoc test for multiple comparisons of mean nucleotide diversity *π* or *θ_w_* between populations of *Rhododendron oldhamii* using Friedman test. The 12 nuclear genes were used as blocking effect.

|  | *π* | |  | *θ_w_* | |
| --- | --- | --- | --- | --- | --- |
| Contrast | Difference | *P* |  | Difference | *P* |
| Between populations | |  |  |  |  |
| BL - CH | 55 | **0.0259** |  | 53.5 | **0.0302** |
| BL - CJ | 62 | **0.0122** |  | 62 | **0.0122** |
| BL - CY | -10.5 | 0.6686 |  | -12.5 | 0.6104 |
| BL - EGS | -15 | 0.541 |  | -11 | 0.6539 |
| BL - HS | -4 | 0.8704 |  | -8.5 | 0.729 |
| BL - HYS | -2 | 0.935 |  | -2 | 0.935 |
| BL - LLK | 3.5 | 0.8865 |  | 3 | 0.9026 |
| BL - LS | -13 | 0.5962 |  | -20 | 0.4152 |
| BL - RL | 31 | 0.2072 |  | 31.5 | 0.2 |
| BL - STS | 40 | 0.1041 |  | 37.5 | 0.1274 |
| BL - TGK | 38.5 | 0.1176 |  | 34.5 | 0.1606 |
| BL - TKL | 57.5 | **0.0199** |  | 56 | **0.0234** |
| BL - WL | 12.5 | 0.6104 |  | 12.5 | 0.6104 |
| BL - WLJ | 35 | 0.1546 |  | 33.5 | 0.173 |
| BL - WR | 4 | 0.8704 |  | 1 | 0.9675 |
| BL - WS | -10 | 0.6835 |  | -7.5 | 0.7598 |
| BL - YP | 3.5 | 0.8865 |  | -2.5 | 0.9188 |
| CH - CJ | 7 | 0.7753 |  | 8.5 | 0.729 |
| CH - CY | -65.5 | **0.0081** |  | -66 | **0.0077** |
| CH - EGS | -70 | **0.0047** |  | -64.5 | **0.0092** |
| CH - HS | -59 | **0.017** |  | -62 | **0.0122** |
| CH - HYS | -57 | **0.021** |  | -55.5 | **0.0246** |
| CH - LLK | -51.5 | **0.0368** |  | -50.5 | **0.0406** |
| CH - LS | -68 | **0.0061** |  | -73.5 | **0.0031** |
| CH - RL | -24 | 0.3284 |  | -22 | 0.3702 |
| CH - STS | -15 | 0.541 |  | -16 | 0.5144 |
| CH - TGK | -16.5 | 0.5013 |  | -19 | 0.4389 |
| CH - TKL | 2.5 | 0.9188 |  | 2.5 | 0.9188 |
| CH - WL | -42.5 | 0.0843 |  | -41 | 0.0958 |
| CH - WLJ | -20 | 0.4152 |  | -20 | 0.4152 |
| CH - WR | -51 | **0.0387** |  | -52.5 | **0.0334** |
| CH - WS | -65 | **0.0086** |  | -61 | **0.0136** |
| CH - YP | -51.5 | **0.0368** |  | -56 | **0.0234** |
| CJ - CY | -72.5 | **0.0035** |  | -74.5 | **0.0027** |
| CJ - EGS | -77 | **0.0019** |  | -73 | **0.0033** |
| CJ - HS | -66 | **0.0077** |  | -70.5 | **0.0045** |
| CJ - HYS | -64 | **0.0097** |  | -64 | **0.0097** |
| CJ - LLK | -58.5 | **0.0179** |  | -59 | **0.017** |
| CJ - LS | -75 | **0.0025** |  | -82 | **0.001** |
| CJ - RL | -31 | 0.2072 |  | -30.5 | 0.2146 |
| CJ - STS | -22 | 0.3702 |  | -24.5 | 0.3185 |
| CJ - TGK | -23.5 | 0.3385 |  | -27.5 | 0.263 |
| CJ - TKL | -4.5 | 0.8544 |  | -6 | 0.8067 |
| CJ - WL | -49.5 | **0.0447** |  | -49.5 | **0.0447** |
| CJ - WLJ | -27 | 0.2717 |  | -28.5 | 0.2461 |
| CJ - WR | -58 | **0.0189** |  | -61 | **0.0136** |
| CJ - WS | -72 | **0.0037** |  | -69.5 | **0.005** |
| CJ - YP | -58.5 | **0.0179** |  | -64.5 | **0.0092** |
| CY - EGS | -4.5 | 0.8544 |  | 1.5 | 0.9512 |
| CY - HS | 6.5 | 0.791 |  | 4 | 0.8704 |
| CY - HYS | 8.5 | 0.7289 |  | 10.5 | 0.6686 |
| CY - LLK | 14 | 0.5683 |  | 15.5 | 0.5276 |
| CY - LS | -2.5 | 0.9188 |  | -7.5 | 0.7598 |
| CY - RL | 41.5 | 0.0918 |  | 44 | 0.074 |
| CY - STS | 50.5 | **0.0406** |  | 50 | **0.0426** |
| CY - TGK | 49 | **0.0469** |  | 47 | 0.0565 |
| CY - TKL | 68 | **0.0061** |  | 68.5 | **0.0057** |
| CY - WL | 23 | 0.3489 |  | 25 | 0.3087 |
| CY - WLJ | 45.5 | 0.0648 |  | 46 | 0.0619 |
| CY - WR | 14.5 | 0.5545 |  | 13.5 | 0.5822 |
| CY - WS | 0.5 | 0.9837 |  | 5 | 0.8385 |
| CY - YP | 14 | 0.5683 |  | 10 | 0.6835 |
| EGS - HS | 11 | 0.6539 |  | 2.5 | 0.9188 |
| EGS - HYS | 13 | 0.5962 |  | 9 | 0.7137 |
| EGS - LLK | 18.5 | 0.451 |  | 14 | 0.5683 |
| EGS - LS | 2 | 0.935 |  | -9 | 0.7137 |
| EGS - RL | 46 | 0.0619 |  | 42.5 | 0.0844 |
| EGS - STS | 55 | **0.0259** |  | 48.5 | **0.0492** |
| EGS - TGK | 53.5 | **0.0302** |  | 45.5 | 0.0648 |
| EGS - TKL | 72.5 | **0.0035** |  | 67 | **0.0068** |
| EGS - WL | 27.5 | 0.2629 |  | 23.5 | 0.3386 |
| EGS - WLJ | 50 | **0.0426** |  | 44.5 | 0.0708 |
| EGS - WR | 19 | 0.4389 |  | 12 | 0.6248 |
| EGS - WS | 5 | 0.8385 |  | 3.5 | 0.8865 |
| EGS - YP | 18.5 | 0.451 |  | 8.5 | 0.729 |
| HS - HYS | 2 | 0.935 |  | 6.5 | 0.791 |
| HS - LLK | 7.5 | 0.7598 |  | 11.5 | 0.6392 |
| HS - LS | -9 | 0.7137 |  | -11.5 | 0.6392 |
| HS - RL | 35 | 0.1546 |  | 40 | 0.1041 |
| HS - STS | 44 | 0.074 |  | 46 | 0.0619 |
| HS - TGK | 42.5 | 0.0843 |  | 43 | 0.0808 |
| HS - TKL | 61.5 | **0.0129** |  | 64.5 | **0.0092** |
| HS - WL | 16.5 | 0.5013 |  | 21 | 0.3923 |
| HS - WLJ | 39 | 0.113 |  | 42 | 0.088 |
| HS - WR | 8 | 0.7443 |  | 9.5 | 0.6986 |
| HS - WS | -6 | 0.8067 |  | 1 | 0.9675 |
| HS - YP | 7.5 | 0.7598 |  | 6 | 0.8067 |
| HYS - LLK | 5.5 | 0.8226 |  | 5 | 0.8385 |
| HYS - LS | -11 | 0.6539 |  | -18 | 0.4633 |
| HYS - RL | 33 | 0.1795 |  | 33.5 | 0.173 |
| HYS - STS | 42 | 0.088 |  | 39.5 | 0.1085 |
| HYS - TGK | 40.5 | 0.0999 |  | 36.5 | 0.1378 |
| HYS - TKL | 59.5 | **0.0161** |  | 58 | **0.0189** |
| HYS - WL | 14.5 | 0.5545 |  | 14.5 | 0.5546 |
| HYS - WLJ | 37 | 0.1325 |  | 35.5 | 0.1489 |
| HYS - WR | 6 | 0.8067 |  | 3 | 0.9026 |
| HYS - WS | -8 | 0.7443 |  | -5.5 | 0.8226 |
| HYS - YP | 5.5 | 0.8226 |  | -0.5 | 0.9837 |
| LLK - LS | -16.5 | 0.5013 |  | -23 | 0.3489 |
| LLK - RL | 27.5 | 0.2629 |  | 28.5 | 0.2461 |
| LLK - STS | 36.5 | 0.1378 |  | 34.5 | 0.1606 |
| LLK - TGK | 35 | 0.1546 |  | 31.5 | 0.2 |
| LLK - TKL | 54 | **0.0287** |  | 53 | **0.0317** |
| LLK - WL | 9 | 0.7137 |  | 9.5 | 0.6986 |
| LLK - WLJ | 31.5 | 0.2 |  | 30.5 | 0.2146 |
| LLK - WR | 0.5 | 0.9837 |  | -2 | 0.935 |
| LLK - WS | -13.5 | 0.5821 |  | -10.5 | 0.6686 |
| LLK - YP | 0 | 1 |  | -5.5 | 0.8226 |
| LS - RL | 44 | 0.074 |  | 51.5 | **0.0368** |
| LS - STS | 53 | **0.0317** |  | 57.5 | **0.0199** |
| LS - TGK | 51.5 | **0.0368** |  | 54.5 | **0.0273** |
| LS - TKL | 70.5 | **0.0045** |  | 76 | **0.0022** |
| LS - WL | 25.5 | 0.2991 |  | 32.5 | 0.1862 |
| LS - WLJ | 48 | 0.0515 |  | 53.5 | **0.0302** |
| LS - WR | 17 | 0.4885 |  | 21 | 0.3923 |
| LS - WS | 3 | 0.9026 |  | 12.5 | 0.6104 |
| LS - YP | 16.5 | 0.5013 |  | 17.5 | 0.4758 |
| RL - STS | 9 | 0.7137 |  | 6 | 0.8067 |
| RL - TGK | 7.5 | 0.7598 |  | 3 | 0.9026 |
| RL - TKL | 26.5 | 0.2806 |  | 24.5 | 0.3185 |
| RL - WL | -18.5 | 0.451 |  | -19 | 0.4389 |
| RL - WLJ | 4 | 0.8704 |  | 2 | 0.935 |
| RL - WR | -27 | 0.2717 |  | -30.5 | 0.2146 |
| RL - WS | -41 | 0.0958 |  | -39 | 0.113 |
| RL - YP | -27.5 | 0.2629 |  | -34 | 0.1667 |
| STS - TGK | -1.5 | 0.9512 |  | -3 | 0.9026 |
| STS - TKL | 17.5 | 0.4758 |  | 18.5 | 0.451 |
| STS - WL | -27.5 | 0.2629 |  | -25 | 0.3087 |
| STS - WLJ | -5 | 0.8385 |  | -4 | 0.8704 |
| STS - WR | -36 | 0.1433 |  | -36.5 | 0.1378 |
| STS - WS | -50 | **0.0426** |  | -45 | 0.0678 |
| STS - YP | -36.5 | 0.1378 |  | -40 | 0.1041 |
| TGK - TKL | 19 | 0.4389 |  | 21.5 | 0.3812 |
| TGK - WL | -26 | 0.2898 |  | -22 | 0.3702 |
| TGK - WLJ | -3.5 | 0.8865 |  | -1 | 0.9675 |
| TGK - WR | -34.5 | 0.1606 |  | -33.5 | 0.173 |
| TGK - WS | -48.5 | **0.0491** |  | -42 | 0.088 |
| TGK - YP | -35 | 0.1546 |  | -37 | 0.1326 |
| TKL - WL | -45 | 0.0677 |  | -43.5 | 0.0774 |
| TKL - WLJ | -22.5 | 0.3594 |  | -22.5 | 0.3595 |
| TKL - WR | -53.5 | **0.0302** |  | -55 | **0.0259** |
| TKL - WS | -67.5 | **0.0064** |  | -63.5 | **0.0103** |
| TKL - YP | -54 | **0.0287** |  | -58.5 | **0.0179** |
| WL - WLJ | 22.5 | 0.3594 |  | 21 | 0.3923 |
| WL - WR | -8.5 | 0.7289 |  | -11.5 | 0.6392 |
| WL - WS | -22.5 | 0.3594 |  | -20 | 0.4152 |
| WL - YP | -9 | 0.7137 |  | -15 | 0.541 |
| WLJ - WR | -31 | 0.2072 |  | -32.5 | 0.1862 |
| WLJ - WS | -45 | 0.0677 |  | -41 | 0.0958 |
| WLJ - YP | -31.5 | 0.2 |  | -36 | 0.1433 |
| WR - WS | -14 | 0.5683 |  | -8.5 | 0.729 |
| WR - YP | -0.5 | 0.9837 |  | -3.5 | 0.8865 |
| WS - YP | 13.5 | 0.5821 |  | 5 | 0.8385 |
|  |  |  |  |  |  |
| Between regions | |  |  |  |  |
| C - N | 3 | 0.6495 |  | -1 | 0.8794 |
| C - S | -2 | 0.7617 |  | -2 | 0.7617 |
| C - SE | -1 | 0.8794 |  | -5 | 0.4501 |
| N - S | -5 | 0.4501 |  | -1 | 0.8794 |
| N - SE | -4 | 0.5451 |  | -4 | 0.5451 |
| S - SE | 1 | 0.8794 |  | -3 | 0.6495 |

*P*, p-values.

*P* < 0.05 are in bold.

**Supplementary Table S6.** Estimated spatial expansion parameters for *Rhododendron oldhamii*.

| Parameter |  | *τ*  (95% CI) | *θ*  (95% CI) | *M*  (95% CI) | SSD  (*P*) | *t* |
| --- | --- | --- | --- | --- | --- | --- |
| Region | N | 35.07  (27.64 - 82.83) | 22.71  (0.01 - 49.75) | 99999  (154.12 - 99999) | 0.00330  (0.796) | 68784 |
|  | C | 24.72  (18.64 - 114.74) | 40.49  (0 - 75.7) | 99999  (106.57 - 99999) | 0.01023  (0.767) | 108768 |
|  | S | 38.59  (30.1 - 86.68) | 29.44  (0.01 - 64.59) | 99999  (152.92 - 99999) | 0.00381  (0.643) | 119685 |
|  | SE | 34.61  (26.71 - 80.53) | 26.76  (0 - 61.52) | 99999  (138.54 - 99999) | 0.00616  (0.658) | 107341 |
|  | Total | 35.96  (18.61 - 128.75) | 32.03  (0.02 - 84.59) | 9239.49  (110.52 - 12284.64) | 0.00123  (0.931) | 111528 |

*τ*: Unscaled expansion time.

*θ*: Unscaled population size.

*M*: Migration rate.

95% CI: Confidence intervals, α = 0.05.

SSD: Sum of squared deviations.

*P*: *P*-value.

*t*: The time at which the expansion event took place.

The time at which the expansion event took place was dated following the expression, *t* = *τ*/2*μ*k, where *τ* is the estimated number of generations since the expansion, *μ* is the mutation rate per site per generation, and k is the sequence length. We assumed a mean generation time of 15 years for *R. oldhamii* in converting the time to expansion to years. Values of *t* estimated according to the mutation rates (*μ*) of 1.581 × 10^-9^ per site per year (Yoichi et al., 2016) are shown, respectively.

**Supplementary Table S7.** Pairwise *F*_ST_ values (below diagonal) and corresponding *P* values (above diagonal) between four regional groups.

|  | North | Central | South | Southeast |
| --- | --- | --- | --- | --- |
| North | - | **0.022** | 0.063 | **0.001** |
| Central | 0.058 | - | **0.027** | **0.003** |
| South | 0.028 | 0.046 | - | **0.001** |
| Southeast | 0.151 | 0.072 | 0.113 | - |

*P* < 0.05 are in bold.

**Supplementary Table S8.** Pairwise comparisons of environmental differences for Taiwania populations occurring in Taiwan based on the eight retained environmental variables using permutational multivariate analysis of variance.

| Pair | *P* value |
| --- | --- |
| North-Central | 0.0045 |
| South-Central | 0.3340 |
| Southeast-Central | 0.0020 |
| North-South | 0.0192 |
| North-Southeast | 0.0020 |
| South-Southeast | 0.0020 |

**Supplementary Table S9.** Values of eight environmental variables at 18 sampling localities of *Rhododendron oldhamii*.

|  | Environmental variables | | | | | | | |
| --- | --- | --- | --- | --- | --- | --- | --- | --- |
| Sites | Aspect | BIO1 | BIO7 | EVI | NDVI | RH | Slope | WSmean |
| BL | 220.1 | 185 | 180 | 0.42 | 0.74 | 79.3 | 31.7 | 2.43 |
| EGS | 338.2 | 192 | 183 | 0.44 | 0.85 | 80.0 | 33.9 | 2.45 |
| HYS | 248.8 | 200 | 186 | 0.45 | 0.81 | 77.3 | 15.9 | 2.96 |
| STS | 10.8 | 184 | 182 | 0.41 | 0.86 | 79.8 | 29.5 | 2.40 |
| TGK | 269.1 | 168 | 185 | 0.42 | 0.83 | 87.4 | 22.7 | 2.94 |
| TKL | 69.4 | 210 | 183 | 0.46 | 0.82 | 79.3 | 20 | 2.62 |
| WLJ | 150.6 | 200 | 188 | 0.46 | 0.87 | 79.7 | 36.5 | 2.42 |
| WL | 265 | 133 | 165 | 0.42 | 0.77 | 78.0 | 30 | 2.20 |
| CH | 170 | 165 | 162 | 0.41 | 0.73 | 78.9 | 15.1 | 3.31 |
| CJ | 172.7 | 149 | 161 | 0.42 | 0.79 | 78.9 | 29.4 | 3.68 |
| CY | 244.4 | 146 | 165 | 0.24 | 0.72 | 77.5 | 40 | 1.77 |
| HS | 285 | 186 | 168 | 0.40 | 0.75 | 78.7 | 33.6 | 2.55 |
| LLK | 302.9 | 147 | 158 | 0.50 | 0.84 | 77.1 | 44.2 | 4.09 |
| LS | 353.3 | 158 | 161 | 0.45 | 0.74 | 78.9 | 28.1 | 3.57 |
| RL | 255.2 | 167 | 159 | 0.44 | 0.82 | 79.7 | 43.7 | 2.34 |
| WS | 124.1 | 175 | 163 | 0.46 | 0.78 | 78.9 | 24 | 3.09 |
| WR | 78.7 | 197 | 157 | 0.45 | 0.81 | 77.3 | 21.7 | 3.02 |
| YP | 95.2 | 161 | 148 | 0.48 | 0.84 | 75.8 | 41.7 | 2.41 |

*BIO1, annual mean temperature; BIO7, temperature annual range; EVI, enhanced vegetation index; NDVI, normalized difference vegetation index; RH, relative humidity; WSmean, mean wind speed.*

**Supplementary Table S10.** Primer sequences and annealing temperatures used in polymerase chain reaction of the 12 genes under study in *Rhododendron oldhamii*.

| Locus | GenBank accession number | Primer sequence (5’-3’) | Annealing temperature (°C) |
| --- | --- | --- | --- |
| *AMP1* | DN161784 | F: CCCTTATTCCCTTGTTGAAGT  R: TCGTATTGGATTTTATGTTATTGTG | 53.5 |
| *ATMYB33* | CV015418 | F: AAACTATTTGAGGCCTGACCTG  R: TCTTCTTCAAAAATGAGTTCCAAA | 60.7 |
| *CPD* | CV014946 | F: CGAGGAGAAAAGGAAAATCAGAG  R: GATTGAAAGTGCGAGCATCC | 61.9 |
| *GRP7* | CV015192 | F: GAACTTGCCGTTTAGCGTTGA  R: CTCAGCCTCCTCAGCCGTAG | 58.3 |
| *PCFS4* | CV015660 | F: CTCTGTTGGGTCCCATAGTC  R: ATTAGGATTAAGGTACAGAGGACCTA | 61.1 |
| *SPA1* | CV015046 | F: GATGGAGTACGAAGAACATGAAAAAC  R: ACCGCGACGTGAATGCTA | 58.1 |
| *GAPC1* | CV015280 | F: CTTTCCGTGTTCCCACTGTT  R: ACCAGGAAACCAACTTCACG | 61 |
| *HEME2* | DN162017 | F: CTGAGCCCCTGTTGCTTA  R: GCCCTTCCCTTTCACAAT | 59.4 |
| *LACS8* | DN161858 | F: AGGTGGAAAAATTGGGGAAA  R: GCCGACGCTTGTATCCAATA | 61 |
| *LHCA1* | DN161900 | F: GTGAGTCCCCAAGTTCTCCA  R: GAACTCATTCACTGCCGATG | 61 |
| *PMDH2* | CV015740 | F: CATGGAAAGTGTTGCAGAGC  R: AAGTTGATGTTCCGGTTGTTG | 61 |
| *SUI1* | CV015175 | F: ATGCTGAGGATTCCAGTGCT  R: GCACTAGGAAGGTGGAGACG | 61 |

Forward (F) and reverse (R) primer pairs for the 12 loci used in the present study were designed using PRIMER3 (http://bioinfo.ut.ee/primer3-0.4.0/) based on expressed sequence tags of *R. catawbiense* (Wei *et al*., 2005, 2006).

**Supplementary Table S11.** Summary of BLASTX results for the 12 loci under study in *Rhododendron oldhamii*.

|  | BLASTX against *Arabidopsis thaliana* protein sequences | | | | | |  | BLASTX against protein sequences of other flowering plants | | | | | | |
| --- | --- | --- | --- | --- | --- | --- | --- | --- | --- | --- | --- | --- | --- | --- |
| Gene | Max score | Query cover | E  value | Identity | NCBI sequence ID | Annotation |  | Max score | Query cover | E  value | Identity | NCBI sequence ID | Annotation |  |
| *AMP1* | 77.1 | 63% | 1e-23 | 55% | NP_567007.1 | Glutamate carboxypeptidase |  | 198 | 78% | 4e-58 | 88% | PSS20837.1 | Glutamate carboxypeptidase [*Actinidia chinensis* var. *Chinensis*] |  |
| *ATMYB33* | 165 | 68% | 5e-45 | 61% | NP_001078537.1 | Myb domain protein 33 |  | 282 | 94% | 1e-93 | 75% | PSS07749.1 | Transcription factor like [*Actinidia chinensis* var. *Chinensis*] |  |
| *CPD* | 303 | 71% | 1e-86 | 76% | NP_001031838.1 | Cytochrome P450 90A1 |  | 304 | 69% | 3e-103 | 87% | AAZ39038.1 | Cytochrome P450 90A2 [*Camellia japonica*] |  |
| *GAPC1* | 377 | 79% | 6e-109 | 94% | NP_187062.1 | Glyceraldehyde-3-phosphate dehydrogenase C subunit 1 |  | 334 | 75% | 5e-119 | 93% | ACY39483.1 | Glyceraldehyde-3-phosphate dehydrogenase [*Panax ginseng*] |  |
| *GRP7* | 61.6 | 46% | 2e-14 | 38% | NP_179760.1 | Glycine-rich RNA-binding protein 7 |  | 146 | 65% | 6e-45 | 75% | XP_021817877.1 | 29 kda ribonucleoprotein A, chloroplastic [*Prunus avium*] |  |
| *HEME2* | 277 | 75% | 6e-79 | 85% | NP_181581.1 | Uroporphyrinogen decarboxylase |  | 278 | 64% | 6e-91 | 89% | XP_023912557.1 | Uroporphyrinogen decarboxylase [*Quercus suber*] |  |
| *LACS8* | 349 | 98% | 2e-100 | 75% | NP_178516.1 | Long chain acyl-coa synthetase 8 |  | 368 | 99% | 1e-125 | 88% | PSS06471.1 | Long chain acyl-coa synthetase [*Actinidia chinensis* var. *Chinensis*] |  |
| *LHCA1* | 398 | 83% | 3e-120 | 91% | NP_001078288.1 | Chlorophyll a-b binding protein 6 |  | 358 | 89% | 7e-124 | 82% | PHT49202.1 | Chlorophyll a-b binding protein 6A, chloroplastic [*Capsicum baccatum*] |  |
| *PCFS4* | 223 | 88% | 8e-63 | 72% | NP_680598.2 | PCF11P-similar protein 4 |  | 244 | 61% | 1e-76 | 86% | PSS10353.1 | Polyadenylation and cleavage factor like [*Actinidia chinensis* var. *Chinensis*] |  |
| *PMDH2* | 207 | 97% | 4e-88 | 93% | NP_001031860.1 | Peroxisomal NAD-malate dehydrogenase 2 |  | 286 | 91% | 1e-95 | 93% | XP_024163549.1 | Malate dehydrogenase, glyoxysomal [*Rosa chinensis*] |  |
| *SPA1* | 251 | 99% | 9e-71 | 50% | NP_182157.2 | Protein SUPPRESSOR OF PHYA-105 |  | 388 | 97% | 6e-136 | 95% | XP_022877928.1 | E3 ubiquitin-protein ligase COP1-like isoform X1 [*Olea europaea* var. *Sylvestris*] |  |
| *SUI1* | 216 | 44% | 8e-61 | 91% | NP_194443.1 | Protein translation factor SUI1 |  | 190 | 39% | 8e-65 | 94% | XP_023876958.1 | Protein translation factor SUI1 homolog 1 [*Quercus suber*] |  |

Max score, the highest alignment score of a set of aligned segments

**Supplementary Table S12.** Variance inflation factor (VIF) and correlation coefficient between the eight retained environmental variables.

|  |  | Correlation coefficient | | | | | | | |
| --- | --- | --- | --- | --- | --- | --- | --- | --- | --- |
| Variables | VIF | Aspect | BIO1 | BIO7 | EVI | NDVI | RH | Slope | WSmean |
| Aspect | 3.033 | 1.00 |  |  |  |  |  |  |  |
| BIO1 | 2.931 | -0.40 | 1.00 |  |  |  |  |  |  |
| BIO7 | 3.971 | 0.25 | 0.49 | 1.00 |  |  |  |  |  |
| EVI | 2.898 | -0.17 | 0.23 | -0.16 | 1.00 |  |  |  |  |
| NDVI | 2.855 | -0.37 | 0.22 | -0.10 | 0.68 | 1.00 |  |  |  |
| RH | 1.469 | 0.23 | 0.12 | 0.52 | -0.16 | -0.13 | 1.00 |  |  |
| Slope | 2.142 | 0.14 | -0.53 | -0.51 | -0.05 | 0.15 | -0.23 | 1.00 |  |
| WSmean | 2.111 | 0.15 | 0.14 | -0.13 | 0.59 | 0.31 | -0.01 | -0.10 | 1.00 |

*BIO1, annual mean temperature; BIO7, temperature annual range; EVI, enhanced vegetation index; NDVI, normalized difference vegetation index; RH, relative humidity; WSmean, mean wind speed.*

**Supplementary Fig. 1.** Genetic clustering results analyzed using discriminant analysis of principal components (DAPC).


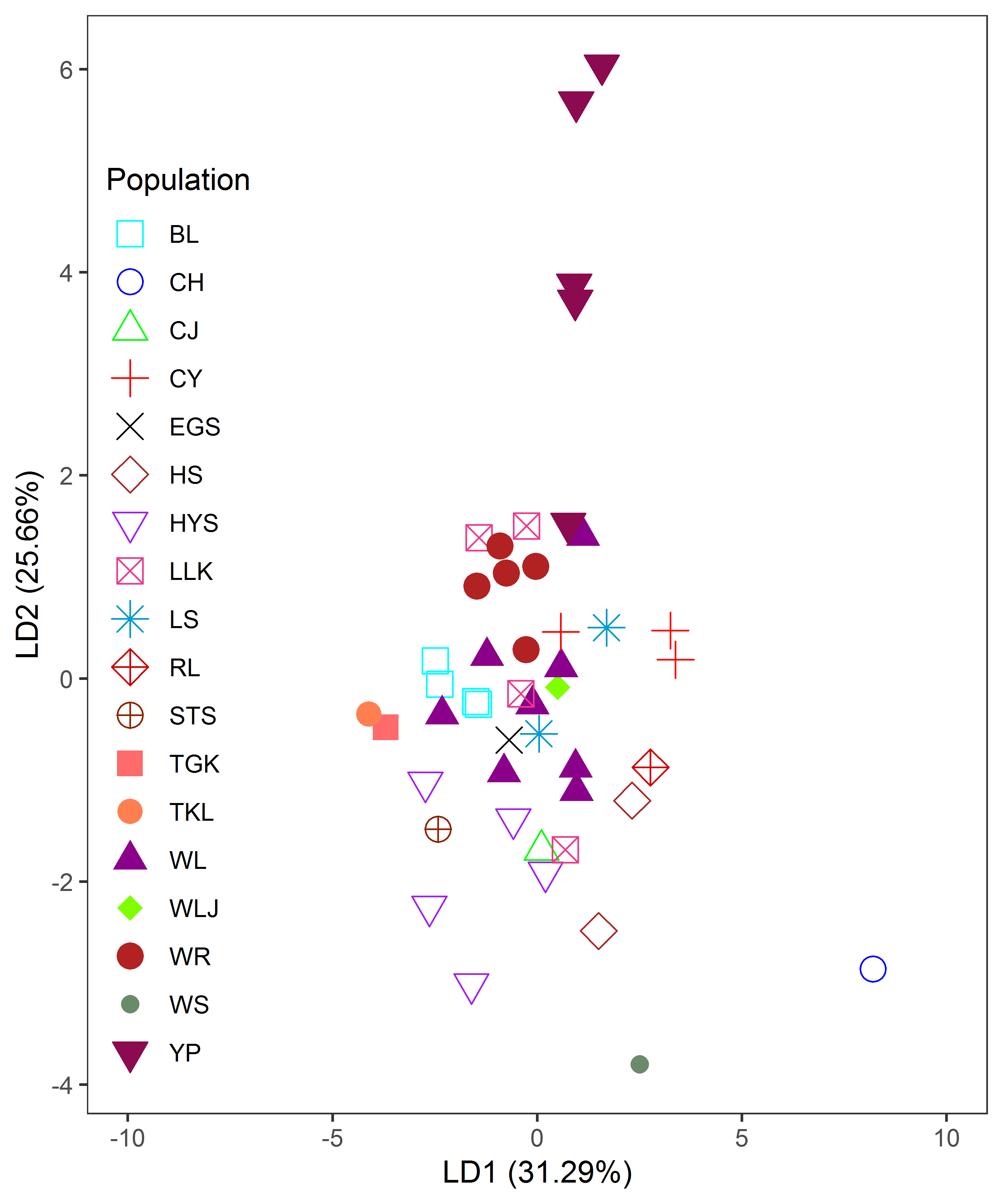

Supplement: Supplementary file 1 — Supplementary information. [file 41598_2020_73748_MOESM1_ESM.docx]
